# Spatial, single-nucleus and pathological profiling of the invasive front in early hepatocellular carcinoma for characterizing specific leading-edge cell niche and improving recurrence modeling

**DOI:** 10.7150/ijbs.137262

**Published:** 2026-09-10

**Authors:** Xicheng Wang, Xiaolan Mu, Yong Fu, Wei Dong, Wenfeng Lu, Xiuhua Li, Yanxiang Song, Changcheng Liu, Baoju Hou, Haibin Zhang, Xiong Chen, Yongchao Cai, Zhiying He

**Affiliations:** 1 Institute for Regenerative Medicine, Medical Innovation Center and State Key Laboratory of Cardiovascular Diseases, Shanghai East Hospital, School of Life Sciences and Technology, Tongji University, Shanghai 200123, China.; 2 Department of Oncology, Mengchao Hepatobiliary Hospital of Fujian Medical University, 350028, Fuzhou, Fujian, China.; 3 Department of Liver Surgery V, The Eastern Hepatobiliary Surgery Hospital, Third Affiliated Hospital of Naval Medical University, Shanghai 200438, P. R. China.; 4 Department of pathology, The Eastern Hepatobiliary Surgery Hospital, Third Affiliated Hospital of Naval Medical University, Shanghai 200438, P. R. China.; 5 Vincent Mary School of Science and Technology, Assumption University of Thailand, Bangkok 10240, Thailand.; 6 Postgraduate Training Base of Shanghai East Hospital, Jinzhou Medical University, Jinzhou, Liaoning 121001, China.; 7 Liver Center and Department of Hepatobiliary Surgery, Ruijin Hospital, Shanghai Jiao Tong University School of Medicine, Shanghai 200020, China.

**Keywords:** tumor leading-edge area (TLE), STMN1-high tumor cells, early HCC, pathology, recurrence

## Abstract

The tumor leading edge (TLE) is a critical region where tumor cells interact with the microenvironment to drive invasion and metastasis; however, its cellular architecture in early hepatocellular carcinoma (HCC) remains poorly understood. Here, we integrated single-nucleus RNA-seq (snRNA-seq), spatial transcriptomics, and computational pathology to investigate TLE in early HCC. We annotated 35 cell subpopulations and identified STMN1-high tumor cells as a key malignant subset enriched at the invasive front, interacting with Treg, plasma B, LAMP3⁺ dendritic cells and SPP1⁺ macrophages. Spatial analysis revealed three co-localized cell pairs—(SPP1⁺ macrophages co-localized with Tip-like and inflammatory endothelial cells), (LAMP3⁺ DCs co-localized with naive T cells), and (plasma B cells co-localized with cancer-associated fibroblasts)—forming a leading-edge tumor microenvironment (L-TME) niche associated with early relapse. We developed an L-TME-related machine-learning benchmark framework incorporating 71 imaging features (65 deep-learning + 6 pathological) based on the snRNA-seq, spatial transcriptomics and pathomics. The pathology model achieved robust performance (mean C-index=0.77) and successfully predicted the recurrence of early HCC (log-rank *p* < 0.05) in TCGA (n=147) and an independent in-house cohort (n=123). This study delineates the TLE cellular ecosystem of early HCC, defines a spatially coordinated immunosuppressive L-TME niche, and provides a clinically applicable predictive tool for postoperative recurrence. Integrating multi-omics with computational pathology deepens our understanding of early HCC metastasis and offers insights into improved prognostication and therapeutic strategies.

## Introduction

Hepatocellular carcinoma (HCC) is a widely prevalent malignant tumor that is recognized as the most common and aggressive form of cancer, with a high global incidence and mortality rate 1, 2. Although liver transplantation, surgical resection, and liver ablation are considered potential treatment options, many patients are deemed inoperable or at risk of recurrence because of local invasion or metastasis. Postoperative recurrence and mortality in 90% of patients are linked to tumor cell metastasis, with the tumor microenvironment playing a critical role in the metastatic process.

The tumor leading-edge region has a complex tumor microenvironment, characterized by hypoxic conditions, intense inflammatory responses, and significant immune evasion 3. Tumor cells can interplay with different cell types within the tumor microenvironment, facilitating tumor progression 4. In the tumor margin region of HCC, some researchers have defined the tumor leading edge as a transitional zone extending 4000-6000 μm (defined as leading-edge area) 5, about 500 μm (referred to as margin area) 6, or about 750-250 μm ("-250 μm to +250 μm" was named as invasive zone) 7 outwards from both sides of the tumor-adjacent interface. Therefore, the width criteria of the leading-edge area could range from 500 μm ("-250 μm, +250 μm") to 1.2 cm ("-6000 μm, +6000 μm"). Importantly, across these studies, the leading edge is consistently described not as a rigid line but as a functional gradient zone where cellular states and interactions gradually transition from the tumor core to the adjacent tissue. Notably, immune evasion was enhanced in tumor cells at the boundary area. Damaged hepatocytes in the invasive zone can secrete SAAs, leading to the recruitment and polarization of macrophages into M2 phenotype via "SAA-TLR2" axis 7, which causes local immune suppression and promotes tumor progression, establishing pro-metastatic niches in the liver 8. Hence, dissecting the tumor microenvironment of HCC and comprehending the cellular characteristics, spatial heterogeneity, and intercellular interactions in the tumor margin region, will assist in elucidating the mechanisms of tumor invasion and metastasis, and aid in the development of novel liver cancer therapies.

Single-cell RNA sequencing (scRNA-seq) and single-nucleus RNA sequencing (snRNA-seq) have emerged as powerful tools for investigating the tumor microenvironment (TME) and dissecting tumor heterogeneity. Current scRNA-seq studies mainly focus on exploring the diversity and heterogeneity of the TME in primary, recurrent, or metastatic HCC 9. Complete characterization of the multicellular ecosystem of early HCC at the single-cell level in the lead-edge area between patients is still lacking 10. Additionally, scRNA-seq/snRNA-seq does not provide spatial information on cells, and because of the lack of sampling from multiple regions, knowledge of single-cell spatial heterogeneity within tumors remains scarce 11. Recently developed spatial transcriptomics (ST) methods enable the generation of single-cell and spatial data from tumor multi-region tissues, which will assist in comprehensive and unbiased tissue analysis to identify intratumoral heterogeneity and TME cell communication, especially in the invasive tumor margin region. Moreover, the development of artificial intelligence, especially machine learning and deep learning algorithms, in deciphering digital data and thereafter enhancing diagnostic and predictive efficiency and accuracy shows great promises 12, 13. However, the combined analysis of understanding early HCC using snRNA-seq, spatial transcriptomics, and computational pathomics requires further investigation.

In the present study, we conducted a comprehensive analysis of the multicellular ecosystem of the tumor TLE zone, including malignant, immune, and stromal cells, to dissect the heterogeneity of tumor microenvironment of the invasive front in early HCC. A gradient-centric analytical framework, was used to show the leading edge, capturing the full transitional dynamics of the leading-edge microenvironment without imposing an overly rigid boundary. For practical spot annotation, we defined a "tumor-to-adjacent" boundary area (TLE_200 μm, the core leading-edge region) spanning -100 μm to +100 μm for high-confidence sampling, while extending gradient analyses to -500 μm to +500 μm—well within the 1.2 cm literature range (Supplementary Table S1)—to observe the full breadth of cellular gradients. Our findings unveil the main immunosuppressive cells in the early HCC TME and emphasize the prognosis-related features of the tumor subpopulation with significantly elevated STMN1 expression. By combining spatial transcriptomic and scRNA-seq/snRNA-seq data, we identified the high cellular and transcriptional heterogeneity in TLE area. Moreover, based upon a multi-omics and pathological framework, we conducted a comprehensive study to characterize a leading-edge TME (L-TME) correlated with high recurrence rates. Our research underscores the pivotal potential of snRNA-seq and spatial transcriptomic approaches in offering valuable insights for the development of new treatment strategies for solid tumors.

## Results

### Single-nucleus RNA-seq analysis reveals the cellular composition landscape of the TLE region in early HCC

Heterogeneity is one of the main challenges in inhibiting tumor recurrence and metastasis. Dissecting the spatial heterogeneity of various cell types and functional cellular states is crucial for the development of effective treatments, especially in the TLE zone, where tumor cell infiltration and invasion are the most active. A flowchart of our study is shown in Figure 1A. The present work was composed of major three aspects: i) Deciphering the cell atlas of early HCC at the leading-edge area based on snRNA-seq and spatial transcriptomics, and exploring the transcriptomic characteristics and profiles of each cell type. ii) Stepwise sub-cluster analysisof each cell types and construction of a prognostic model for whole-stage HCC by combining the scRNA-seq, bulk RNA-seq, snRNA-seq and spatial transcriptomics validated in multiple cohorts. iii) Development of a relapse pathological model for early HCC based on digital pathology, snRNA-seq and spatial transcriptomics, to aid in determining the recurrence of early HCC after surgery.

With the aim of achieving the aforementioned three major aspects, we firstly collected early HCC samples from the TLE region for single-nucleus RNA sequencing (snRNA-seq) (five early HCC patients from our center for snRNA-seq and 123 patients with early HCC from our center for validation) (Figure 1A). By conducting initial single cell clustering and celltype annotation of the snRNA-seq data, we identified hepatocyte/hepatocellular carcinoma cell populations (parenchymal cell populations), immune cell populations, stromal cell populations, endothelial cell populations, and myeloid cell populations (Figure 1B and 1C). Furthermore, we performed spatial transcriptomic sequencing on early HCC samples from the TLE region (4 early HCC patients from our center for spatial transcriptomic sequencing and 123 patients with early HCC from our center for validation) (Figure 1A). The overall quality control results of the spatial transcriptomic data are shown in Figure S1A-S1C. For identifying the "tumor-adjacent" interface of early HCC, we utilized the morphology (H&E images), expression data and inferred CNV score data to detect the potential boundary spots. More, >=2 pathologists assessed and categorized the tumor region, boundary region, and adjacent region of the H&E pictures of the spatial transcriptomic data, as well as all other 123 early HCC patients (Figure 1D and Figure S1D). With the reassurance of the manual selection of boundary spots after obtaining the computed non-malignant, boundary and malignant spots, highly confident results were collected. The spot width of 10× Visium technology is 50-550 μm, and the distance from one spot to another spot is 100 μm (https://www.10xgenomics.com/cn/platforms/visium). Thus, the identified boundary spots could be named as TLE 0-200 μm, indicating the boundary spots were included in the 200 μm width from "tumor-interface-adjacent" (-100, 0, +100 μm). Based on the identified boundary spots (TLE: 0-200 μm), we further calculated the potential layers (100 μm per layer to the interface of "tumor-adjacent") (Figure 1D).

Furthermore, we performed detailed annotation of the cell subpopulations for each cell group (Figure S2A and S2B). Specifically, it can be observed that (1) Parenchymal cells were categorized into benign and malignant cells, with malignant cells further divided into 14 subgroups, suggesting considerable heterogeneity in malignant cells. (2) B cells were mainly categorized into B cells and Plasma B cells. (3) T/NK cells were mainly divided into CD56^bright^ NK cells, CD56^dim^ NK cells, Th17 cells, Naive T cells, Tem cells, CTL cells, and Treg cells. (4) Monocytes were predominantly categorized into CD14^+^ and CD16^+^ monocytes. (5) Dendritic cells were mainly divided into four subgroups: CLEC9A^+^ DC1, CD1C^+^ DC2, LAMP3^+^ DC3, and CLEC4C^+^ pDC cells. (6) Macrophages were primarily classified as APOE^+^ macrophages, CXCL10^+^ macrophages, MACRO^+^ macrophages, and SPP1^+^ macrophages. (7) Stromal cells primarily included cancer-associated fibroblasts (CAFs), vascular smooth muscle cells (VSMCs), pericytes, liver sinusoidal endothelial cells (LSECs), inflammatory ECs, lymphatic ECs, tumor-associated EC (TECs), arterial ECs, and Tip-like ECs.

Based on the above analysis, the cell types in the TLE region of early HCC were annotated. To understand the relationship between the cell types distributed at the tumor boundary and the clinical prognostic outcomes of patients with HCC, we explored their potential correlation with HCC prognosis. Because the number of death events in our early-stage cohort was insufficient to train a robust prognostic model for overall survival, and because the cell types identified at the early-stage were also present in late-stage malignancies, possibly differing mainly in abundance rather than in lineage 14. Furthermore, the leading edge is the site where invasion and immune modulation occur, and its cellular composition may capture intrinsic tumor aggressiveness that is not strictly stage-dependent 3. We therefore tested whether a deep learning model trained on the 35 early-stage-derived cell types could stratify patients with whole-stage HCC. The DeepSurv Python package (version 0.4.1) was used to construct a survival model based upon cellular composition features, to investigate the correlation between the cell types in the invasive front of HCC and patient prognosis 15. As shown in Figure S2B, we employed a two-step deconvolution-based approach to construct a prognostic model. Using our leading-edge snRNA-seq data, we defined 35 cell-type-specific gene expression signatures as crucial references. Then, BayesPrism was used to deconvolute the bulk RNA-seq data (TCGA-LIHC and external cohorts) to estimate the relative abundance of each leading-edge-derived cell type within each bulk tumor sample. Therefore, the DeepSurv prognostic model was trained using the inferred abundances of the 35 cell types in the bulk RNA-seq dataset of whole-stage HCC. The results indicated that this tumor boundary-based deep learning model could predict the prognosis of patients with whole-stage HCC (Figure S2B and S2C), and it was validated in multiple external cohorts, including ICGC-LIRI-JP, GSE14520, GSE124751, OEP000321 and GSE148355, yielding strong predictive performance (Figure S2B and S2C). Therefore, the results (Figure S2C-S2D) support this possibility that the cell types from the leading-edge area of HCC were highly related to the prognosis of HCC, but we acknowledged its exploratory nature due to the source of cell types only from early HCC. Moreover, it also indicated the crucial role of the cell types in the leading-edge area of early HCC (Figure S2B and S2C) and it is crucial to thoroughly explore each cell type identified in the TLE region.

### Heterogeneity of malignant cells: STMN1-high subpopulations exhibit distinct spatial distributions at the tumor invasive front

Having established the overall landscape of cell types in the leading-edge area, we next performed in-depth sub-clustering of each major lineage to identify functionally distinct subpopulations. Initially, we classified the parenchymal cell subpopulations using snRNA-seq and identified two hepatocyte clusters and 12 tumor cell subpopulations (Figure 2A). We then employed a bio-pathway-based multi-gene regression method (scPagwas), performing multi-gene linear regression between pathway scores based on scRNA-seq data and GWAS genetic signals to infer a set of trait-associated genes for identifying trait-related subpopulations 16. Here, the tumor cells exhibited a higher scPagwas score than the hepatocytes (Figure 2B). Additional cell cycle scoring analysis indicated that the three tumor cell groups demonstrated enhanced proliferative capacity (Figure 2C). Differential gene expression (DEG) analysis revealed that tumor cells in the TLE zone of early HCC highly expressed STMN1 (Figure 2D). Further analysis of the tumor cell subpopulations identified three tumor cell groups with markedly higher STMN1 expression (STMN1-high subpopulations), with STMN1-high (C11) showing the highest STMN1 expression levels (Figure 2E). We therefore collectively termed these three clusters as "STMN1-high tumor cells" for analyses that assessed the combined effects of STMN1-high malignant cells. Where a specific cluster possessed unique functional features, we referred to it individually. Notably, relative to STMN1-high (C7 and C10), STMN1-high (C11) tumor cells exhibited stronger proliferative capacity, increased procoagulant activity, and activation of pathways such as MTORC, KRAS, and MYC, which are well-known to be capable of driving tumor cell progression (Figure 2F). Moreover, the SCENIC pipeline was leveraged, and the results indicated that STMN1-high tumor cells were dominantly regulated by E2F family members, including E2F1, E2F2, E2F7 and E2F8, based on both gene expression levels and regulon activities (Figure S3).

To validate the results of snRNA-seq data, we used spatial transcriptomic and scRNA-seq data to performed an integrative analysis, highlighting the crucial role of STMN1-high tumor cells in early HCC. Specifically, spots detected by 10× Visium technology usually contained 1-10 cells, making it necessary and reciprocal to perform integrative analysis with scRNA-seq. Therefore, scRNA-seq data of a total number of 88 tissues from 34 patients with whole-stage HCC from three different cohorts were collected and 149392 cells were obtained 17-19 (Figure S4A-E). As shown in Figure S4A and S4B, all cells were separated into four major subpopulations: parenchymal/tumor cells, stromal cells, lymphoid cells and myeloid cells. The four subpopulations were further regrouped into 36 clusters and 14 cell types (Figure S4B and S4C). According to the principal component analysis (PCA), the distinct parenchymal and mensenchymal branches existed in all 34 patients (Figure S4D and S4E), and the proportion of different cell types varied among 34 patients, 88 tissues, and four tissue sources (lymph node, non-tumor, PVTT and tumor tissue) (Figure S4F). These clusters were used to annotate the spatial regions of whole-stage HCC tissues based on canonical correlation analysis (CCA) 20. Surprisingly, most spots in the leading-edge region of early HCC were annotated as benign or tumor cells, whereas a few immune cells were identified, within the tumor area (Figure S4G and S4H). We also found that CD45 was mainly expressed in adjacent tissues rather than in tumor tissues, indicating a potential crosstalk in the leading-edge area (Figure S4I).

Combined analysis of spatial transcriptomics and scRNA-seq enabled the mapping of cell types to the leading-edge region. We explored the spatial annotation result of all 36 single-cell clusters obtained from 34 patients with whole-stage HCC. Interestingly, cluster cl_05 tended to be located in the inner tumor area rather than in the leading-edge region. In contrast, cl_22 was localized in the outer regions of the tumors. Meanwhile, cl_27 cells clustered around regions full of blood vessels/endothelial cells (Figure S5A). According to the stacked graph shown in Figure S5B, cl_05 and cl_22 were single cells mainly obtained from tumor tissues, which was consistent with the predicted spatial information. In contrast, most cl_27 were dissociated from PVTT tissues, reasonably explaining their endothelial/mesenchymal characteristics, which might be acquired during the vascular invasion and metastasis via the EMT pathway. We then overlapped their differentially expressed genes and found that only 31 genes were upregulated among these three clusters (Figure S5C).

As exhibited in Figure S5C, we found that only STMN1 was gradually up-regulated along the "cl_05 -cl_22 -cl_27" axis, representing that the spatial expression of STMN1 showed an upward tendency from the inner tumor, outer tumor to the vascular endothelium region (Figure S5D and S5E). Therefore, we named these three space-characterized cell subclusters STMN1-high tumor cell clusters and considered STMN1-high tumor cells might participate in the vascular invasion process of HCC. We further attempted to understand the expression pattern of STMN1 in the leading-edge region using spatial transcriptomic data. Interestingly, the spatial transcriptomic data indicated that STMN1 was highly expressed not only in the tumor tissue but also in the MVI region (Figure S5F and S5H). The MVI was also highly expressed CD34 (Figure S5G and S5I). We postulated that STMN1-high tumor cells also existed at the MVI, micro-emboli, and macro-emboli (PVTT), playing a crucial role in initiating and promoting vascular invasion from non-invasive early HCC to aggressively invasive advanced HCC. We then collected the leading-edge region, which contained both the ongoing and complete processes of tumor cells invading vascular vessels. Concordantly, STMN1 expression was higher in the tumor and tumor foci area, rather than in the normal area, and STMN1 was highly expressed in tumor cells, especially in the inside small vessels (Figure S5J).

In summary, spatially resolved STMN1-high tumor cells play a pivotal role in vascular invasion from the inner tumor area, outer tumor area, and small vessels (MVI) to large vessels (PVTT), thereby contributing to the progression of early HCC to advanced HCC.

### Validation of the crucial prognosis-related roles of STMN1-high tumor cells in several external cohorts

Additionally, we applied the TCGA public database to assess the expression of these cell groups and observed that the three STMN1-high tumor cell subpopulations were significantly upregulated in whole-stage HCC tissues (Figure 2G). Further integration of more than 10 databases yielded consistent results (Figure 2H). To confirm that STMN1-high tumor cells were enriched in the TLE region, we performed a position analysis (distance to the boundary) of the spatial transcriptomic data. Intriguingly, STMN1-high tumor cells were enriched in the TLE area (mainly distributed at "-300 μm to 100 μm" of the TLE region) (Figure 2I). Consistent with the position analysis, immunohistochemical assays further demonstrated that STMN1 expression was higher at the tumor margin than in the tumor interior area (Figure 2J). We performed semi-quantitative scoring of STMN1 IHC on all 123 in-house early HCC cases. The results showed that in 89/123 cases (72.4%) , STMN1 expression was relatively higher in the TLE area than in the tumor core, confirming the pattern observed in Figure 2J. In the other 34/123 (27.6%) patients, STMN1 expression was similarly low in both the tumor and TLE area. Moreover, survival analysis indicated that these three cell groups were strongly associated with an adverse prognosis (Figure 2K). Interestingly, we found that three survival curves appear similar due to the high-/low-risk group classifications based upon the three STMN1-high subpopulations were highly overlapping due to their correlated abundances (Figure S6A). High STMN1 expression was associated with poor prognosis (Figure 2L). Consistently, the enrichment of the three STMN1-high tumor cells also resulted in a worse prognosis (Figure S6B and S6C). The survival analyses of the three STMN1-high tumor cells (C7, C10 and C11) were quite similar, indicating that all of them were up-regulated in tumor tissues and could affect the prognosis of patients with HCC, which was consistent with the expression pattern of STMN1 gene expression. Therefore, in the following sections, we denoted them as STMN1-high tumor cells and considered them crucial prognosis-related risk factors.

We then performed the CNV analysis for the spatial transcriptomics of early HCC (Figure S7A and S7B). The malignant area was correctly identified by CNV analysis (Figure S7C and S7D). The deconvolution results for the spots were in line with the CNV results (Figure S7E). The deconvolution results also demonstrated that the immune cells were enriched in the boundary and non-malignant area (Figure S7F). Then, we performed the sub-spot analysis and obtained the pseudo-scRNA-seq of spatial transcriptomic data. Concordantly, STMN1 was highly expressed in tumor cells in the boundary area compared to those in the malignant area (Figure S7G and S7H), concordant with the aforementioned results of position analysis. Parallelly, the scRNA-seq data of HCC with or without MVI were also obtained for validation (Figure S7I and S7J). STMN1 was consistently and highly expressed in MVI-positive tumor cells (Figure S7K).

In summary, we discovered that tumor cells at the TLE region (especially at "-300 to 100 μm") exhibited high STMN1 expression, with three STMN1-high tumor cell subpopulations identified, all closely associated with poor prognosis.

### Plasma B cells enriched in the TLE area may be a key cell subpopulation in the liver cancer TLE region

We also identified the leading-edge area of the spatial transcriptomic data as T1, T2 and T3 (Figure S8A) 21 and spatial pseudotime analysis was conducted using the stlearn package (Figure S8A). Interestingly, results showed that following the trajectory from inner tumor area to outer tumor area, immune-related genes, including immune-related markers covering antigen presentation (HLA-A, HLA-B, HLA-E, HLA-DQB1, HLA-DQA1, CD74), complement components (C3, C1QA, C1QB, C1QC, C1R, CFB, SERPING1, CLU), acute-phase response proteins (HP, SAA1/SAA2, SERPINA1, SERPINA3, ORM1, LBP, AHSG, ITIH3/ITIH4), chemokines and adhesion molecules (CCL21, LTB, ICAM1), S100 calcium-binding proteins (S100A4/A6/A9/A10/A11), leukocyte markers (CD52, CD63, LYZ, CTSB), and mesenchyme-related genes, comprising the ECM-related genes include structural components (COL1A1, FGA, FGB, FGG, FGL1, DCN, ITIH3, ITIH4, AHSG, CLU, AMBP), ECM-remodeling proteases and inhibitors (TIMP1, SERPINE1, CTSB, CTSD, SERPINA1, SERPINA3), as well as adhesion molecules (ICAM1, ITGB2, CD63, TM4SF4, IGFBP2) were gradually enriched (Figure S8B). Therefore, cell types other than parenchymal cells, also played crucial roles in the leading-edge area of early HCC. Thereafter, we conducted in-depth sub-clustering of immune and stromal cells to investigate the functional subpopulations.

Furthermore, we conducted an in-depth clustering annotation of B cell subpopulations. The B cell subpopulations were classified into two groups: canonical B cells and plasma B cells. Additionally, we performed scPagwas scoring and cell propensity allocation scoring analyses, revealing that plasma B cells exhibited higher scPagwas scores, suggesting their significant role in the invasive region (Figure 3A-D). Further enrichment analysis indicated that plasma B cells might be closely associated with the endoplasmic reticulum stress phenotype, suggesting that plasma B cells undergo endoplasmic reticulum stress in the TLE area, which may lead to functional impairment of plasma B cells (Figure 3E-F). Correlation analysis further indicated a significant association between scPagwas scores and ERS, with the detection of ERS frequently corresponding with higher scPagwas scores (Figure 3G). Cell-cell communication analysis and immunofluorescence staining also indicated that plasma B cells might communicate with STMN1-high tumor cells in the TLE area (-100 μm, +100 μm) (Figure 3H-I). Concordantly, metabolic analysis also indicated that the metabolism state was more active in plasma B cells in the TLE area than in B cells (Figure 3J). In summary, plasma B cells might experience endoplasmic reticulum stress in the TLE region, forming an immunosuppressive microenvironment, which in turn facilitates the metastasis of STMN1 tumor cells, positioning them as a crucial cell subpopulation in the liver cancer boundary region 22.

Considering that Plasma B cells may interplay with other immune cell types, we initially applied deconvolution to the spatial transcriptomics data to calculate the possible spatial infiltration levels of different cell types. Correlation analysis revealed a strong correlation between CAF and plasma B cell infiltration levels (Figure S9A). We performed spatial co-localization analysis utilizing the SpaCET package (Spatial Cellular Estimator for Tumors) in R 23. Interestingly, the results suggested a significant interaction between CAF and plasma B cells (R=0.64, P-value<2.2e-16) (Figure S9B-E).

### Identification of T cell subpopulations in the immunosuppressive TME at the leading-edge area of early HCC

Subsequently, we performed an in-depth subpopulation annotation of T/NK cells. Seven subpopulations were identified: CD56^bright^ NK cells, CD56^dim^ NK cells, Th17 cells, Naive T cells, Tem cells, CTL cells, and Treg cells (Figure 4A and 4B). Furthermore, scPagwas scoring analysis revealed higher scores for Naive T cells and Treg cells (Figure 4C and 4D). Moreover, with an increase in scPagwas scores, the T cell function-related scores were markedly reduced (Figure 4E and 4F). Interestingly, we observed that Naive T cells and Treg cells expressed several immunosuppression-related genes (Figure 4G and 4H). Correlation analysis also showed that these two cell populations were more similar (Figure 4I). Further cell-cell communication analysis also showed that Naive T cells and Treg cells communicated more actively with STMN1-high tumor cells (Figure 4J and 4K). Multiplex immunofluorescence staining showed that FOXP3 not only expressed in the nuclear area of Treg cells, but exhibited both in the nuclear and cytoplasmic area in a subset of tumor cells, consistent with previous reports in HCC 24-26. This further confirmed the close positioning and interaction between Treg cells (CD3^+^FOXP3^+^) and STMN1-high tumor cells in the TLE zone (Figure 4L).

Through metabolic analysis, we found that the metabolism of Naive T cells and Treg cells was more active (Figure S11A-D). It is noteworthy that the spatial transcriptomics data suggested the significant activation of various pro-tumor signalling pathways in Naive T cells, particularly the TNFa signalling pathway (Figure S11E). Intriguingly, consistent with our findings of increased Naive T cells in the liver cancer junction zone, literature has also reported a significant increase in the proportion of Naive T cells in HCV-HCC 27, highlighting that during hepatitis C virus infection, Naive T cells increase but rarely differentiate into mature stages. This may be a key link in HCC viral infection, leading to HCC. Overall, Naive T cells and Treg cells might represent critical cell subpopulations that played a key role in the immunosuppressive microenvironment of the early HCC "tumor-nontumor" junction zone.

Given that the proportion of cells expressing Treg markers appeared to be low, several related analyses were conducted to further confirm the annotation of Treg clusters. We performed linear dimensionality reduction, PCA, on all T/NK cells and re-examined the distribution of Treg cells. The Treg clusters remained distinctly separated from the other T/NK cell subsets, and no obvious subclusters were observed within the Treg population (Figure S11F). Differential expression analysis between Treg cells and all other T/NK cells revealed that CTLA4, a canonical Treg marker, was highly and uniformly expressed in the Treg subpopulation (Figure S11G). We estimated a "Treg score" using the "AddModule" function in Seurat, established on the core markers FOXP3, IL2RA, CTLA4, IKZF2, and TNFRSF4. As shown in the violin plot (Figure S11H), the Treg score was the highest and uniformly distributed in the Treg cluster. Pseudotime analysis was conducted on T/NK cells using scTour analysis 28. The results showed a continuous differentiation path from naïve T cells to Treg cells, with Treg cells localized at the terminal end of the trajectory (Figure S11I). No additional branches or isolated clusters were observed within the Treg lineage, supporting a genuine differentiation origin rather than contamination from unrelated cell types.

### Myeloid cell components in the leading-edge area of early HCC: LAMP3^+^ dendritic cells were spatially co-localized with Naive T cells and Treg cells

Moreover, we conducted a detailed subgroup annotation of the myeloid cell subsets. The main subgroups were dendritic cells, macrophages, and monocytes (Figure S12A-C). Additionally, we further categorized DCs into four types (Figure 5A-5B). Given that DCs often facilitate the establishment of an immunosuppressive microenvironment by recruiting T cells 29, we further conducted a co-localization analysis and found that DCs co-localized with Naive T cells (Figure S12D), indicating a close relationship between DCs and Naive T cells. Consistently, we conducted a cell ratio correlation analysis, which revealed a close relationship among LAMP3^+^ DCs, Naive T cells and Treg cells (Figure 5C). In parallel, literature suggests that in liver cancer, DCs can regulate the activation and recruitment of Naive T cells 30. Functional scoring analysis of DCs revealed that LAMP3^+^ DCs had higher migration and activation scores (Figure 5D). Moreover, LAMP3^+^ DCs expressed lower levels of MHC (Figure 5E), indicating that they may play crucial roles in the immunosuppressive microenvironment at the early HCC boundary. GSVA enrichment analysis revealed that LAMP3^+^ DCs were more active, with a higher number of activated pathways (Figure 5F). Next, we performed a metabolic analysis on the four types of dendritic cells, which suggested that LAMP3^+^ DCs had a more active state of cell metabolism (Figure S12E and S12F). Our results also indicated that LAMP3^+^ DCs prominently expressed CD274 (Figure 5G). Immunofluorescence staining demonstrated that LAMP3^+^ DCs might be capable of recruiting Treg cells at the early HCC boundary (Figure 5H), which aligned with their high tolerance scores, suggesting their multifunctionality and immunosuppressive characteristics. LAMP3^+^ DCs had high CD274 (PD-L1) expression, which has been reported to be able of recruiting T cell subsets to the tumor as well as modulating the TME 29, 31. In summary, LAMP3^+^ DCs may contributed to the creation of an immunosuppressive microenvironment by recruiting Naive T cells and Treg cells.

### Myeloid cell composition in tumors: The close relationship between SPP1^+^ macrophages and STMN1-high tumor cells

Next, we clustered the macrophages and identified four distinct groups: APOE^+^ macrophages, CXCL10^+^ macrophages, MACRO^+^ macrophages, and SPP1^+^ macrophages (Figure 5I and J). Interestingly, the APOE^+^ macrophages, which are lipid-associated macrophages, were relatively inactive (Figure S13A). Further scoring of M1 and M2 phenotypes revealed that the M1 phenotype was the most prominent in CXCL10^+^ macrophages, indicating an underlying pro-inflammatory phenotype (Figure S13B). Furthermore, a previous study has suggested that SPP1^+^ macrophages interact with CAFs to limit immune infiltration and promote tumor progression 32. This suggests that the SPP1 signalling pathway was a important pathway in the immunosuppressive microenvironment of early HCC (Figure 5K and Figure S13C). In contrast, the metabolic analysis showed that the metabolism flux of SPP1^+^ macrophages was the most active (Figure 5L and S13D). Cell communication and spatial cell communication analyses revealed that the relationship between SPP1^+^ macrophages and STMN1-high tumor cells was the closest, with the "SPP1" signalling pathway being highly active (Figure 5M). Multiplex immunofluorescence staining further confirmed the close interaction between STMN1-high tumor cells and SPP1^+^ macrophages in the TLE region (Figure 5N).

Subsequently, we classified the monocytes into two groups: CD14^+^ monocytes and CD16^+^ monocytes (Figure S14A). Interestingly, spatial cell co-localization analysis revealed that CD14^+^ monocytes had a higher infiltration level than CD16^+^ monocytes (Figure S14B and S14C), and were highly active. Further cellular co-localization analysis indicated that CD14^+^ monocytes were located very closer to CAFs than CD16^+^ monocytes (Figure S14D), suggesting that CD14^+^ monocytes might interact with CAFs in the early HCC TLE area (Figure S14C-D).

### Dissection of stromal cell components in the early HCC TLE region: annotation of stromal cells and endothelial cells

On the other hand, we conducted a detailed annotation and analysis of stromal cells, identifying a total of nine cell subgroups. Clearly, Pericytes, CAFs, and VSMCs exhibited stromal cell characteristics, while the others represented endothelial cell phenotypes: liver sinusoidal endothelial cells (LSECs), inflammatory ECs, lymphatic ECs, tumor-associated EC (TECs), arterial ECs, and Tip-like ECs (Figure 6A-B). Correlation analysis also revealed a stronger correlation among endothelial cells and stromal cells, respectively (Figure 6C). Undoubtedly, we discovered that CAFs exhibit more pro-cancer functions, such as TGFb signalling, ECM, MMPs, and others (Figure 6D). Several reported CAF subgroups were collected, and we found that our CAF annotation closely aligned with these subgroups (Figure 6E), confirming the accuracy of our annotation. Intriguingly, metabolic analysis revealed that CAFs exhibited the most active metabolism (Figure 6F and 6G). Subsequently, we performed cell communication analysis and observed that most cell subgroups had more intense interactions with tumor cells than with hepatocytes (Figure 6H). Notably, multiplex immunofluorescence confirmed that the main interaction in stromal cells was between CAFs and STMN1-high tumor cells (Figure 6H and 6I). Further spatial signalling pathway co-localization analysis showed that CAFs activated more pathways, including the TGFb signalling pathway (Figure 6J). In conclusion, CAFs might play a significant role in interacting with STMN1-high tumor cells, facilitating their progression and metastasis.

Next, we conducted a spatial co-localization analysis of endothelial cells, and the results indicated that arterial endothelial cells have the most significant co-localization with pericytes, which is consistent with physiological knowledge (Figure S15A). Furthermore, given that macrophages are typically recruited from the blood, we focused on the relation between macrophages and endothelial cells. Interestingly, SPP1^+^ macrophages were found to be closely associated with endothelial cells (Figure S15B), particularly inflammatory endothelial cells and Tip-like endothelial cells (Figure S15C). Cell metabolic analysis also suggested that the metabolic states of inflammatory endothelial cells and Tip-like endothelial cells were more similar with each other, whereas lymphatic endothelial cells exhibited highly active metabolism (Figure S15D and S15E). In addition, spatial signalling pathway co-localization analysis of endothelial cells revealed that TECs activated more tumor-related signalling pathways, which aligned with the cell annotation results. Overall, endothelial cells at the TLE region exhibited substantial heterogeneity and were closely associated with macrophages.

Considering that CAFs may elevate the infiltration of other immune cell types, we performed a co-localization analysis of all cell types utilizing the mistyR package. Similarly, a strong co-localization between CAFs and SPP1^+^ macrophages was observed, as well as between plasma B (Figure S16A). Additionally, upon analyzing the specific contribution of cell-cell communication, we observed that pericytes primarily engaged in direct contact (Figure S16B), which was consistent with the characteristic localization of pericytes around blood vessels. Therefore, the results of the mistyR analysis were consistent with earlier findings from the SpaCET package, where strong co-localization between plasma B cells and CAFs was observed. Additionally, using the cell degree package for further analysis, we found that CAFs, SPP1^+^ macrophages and plasma B cells were mainly co-localized in the tumor TLE region (Figure S16C and S16D).

### Identification of the unique co-localized community of TME at the tumor TLE zone of early HCC

In summary, we have outlined the characteristics of specific cell types identified using snRNA-seq and spatial transcriptomics. To gain a comprehensive understanding of the co-localized associations between various stromal and immune subpopulations in the TLE zone of early HCC, we focused on the joint analysis of snRNA-seq and spatial transcriptomics. Therefore, the spots obtained from spatial transcriptomics were deconvoluted to derive the potential scores of each cell type at spot levels using a proportional correlation analysis of these cell types. We observed that pericytes were clustered together with arterial endothelial cells, and tumor cells also tended to aggregate together. More interestingly, we found that Naive T cells and LAMP3^+^ DCs also clustered together, which was in line with the previous snRNA-seq results of the expression analysis (Figure 5C and 7A). Specifically, the co-localization analysis showed that (i) Plasma B cells co-localizied with CAF, (ii) LAMP3^+^ DC3 co-localized with Naive T cells, (iii) SPP1^+^ Macrophages co-localized with Tip-like EC and inflammatory ECs. These three co-localized pairs, especially Plasma B cells, CAF, LAMP3^+^ DC3, Naive T cells and SPP1^+^ Macrophages, also exhibited potentially distinct functions compared to other corresponding cell types in the aforementioned sub-cluster analyses (Figure 3-6). Thus, they might collectively constitute a spatially coordinated multicellular functional community in the TLE region, which we defined as the "leading-edge tumor microenvironment (L-TME)" niche. The results revealed that the L-TME niche was highly enriched in tumor tissues (Figure 7B) and was tightly associated with prognosis (Figure 7C).

Furthermore, we observed that CAFs, the well-studied drivers of tumor invasion and metastasis, co-localized with plasma B cells in the early HCC leading-edge area (Figure 7D-7G and Figure S9). More importantly, the spots where they interacted were clearly closer to the tumor margin (Figure 7D-7E). Position analysis indicated that CAFs and plasma B cells were both enriched at TLE 0-200 μm ("tumor-interface-adjacent ", "-100 to +100 μm") area (Figure 7F). Considering that DCs often facilitate the establishment of an immunosuppressive microenvironment by recruiting T cells 29, this co-localization analysis also confirmed that LAMP3^+^ DCs co-localized with Naive T cells (Figure 7H-J), indicating a close relationship between LAMP3^+^ DCs and Naive T cells. Position analysis indicated that LAMP3^+^ DCs and Naive T cells were both enriched at the TLE ("-100 to +300 μm") area (Figure 7H).

Furthermore, we discovered that SPP1^+^ macrophages clustered with two types of endothelial cells, suggesting that SPP1^+^ macrophages might originate from the blood rather than from liver-resident macrophages (Kupffer cells) (Figure 7K-M). Spatial co-localization analysis also indicated that SPP1^+^ macrophages strongly co-localized with inflammatory endothelial cells (Figure 7A). We also found that the abundance of SPP1^+^ macrophages, Tip-like ECs and inflammaging ECs was higher in the TLE 0-200 μm region compared to the tumor and adjacent regions (Figure 7K). Position analysis also indicated that SPP1^+^ macrophages, Tip-like ECs and inflammaging ECs were enriched in TLE ("0 to +400 μm") (Figure 7M). Moreover, SPP1^+^ macrophages also co-clustered with plasma B cells and CAFs, indicating the co-localization of these three cell types, as validated by spatial transcriptomics and immunofluorescence assays (Figure S16C, Figure S17). Collectively, in line with the earlier cell subpopulation analysis, these clustered subpopulations (L-TME niche) likely played a pivotal role in the immunosuppressive microenvironment of the TLE region in early HCC. The L-TME niche was spatially confined to the tumor-adjacent interface, predominantly within 400 μm of the histologically defined boundary, straddling both the outermost tumor cell layers and the immediately adjacent stromal/inflammatory region (Figure 7D-7F)."

### A L-TME-based recurrence predictive model of early HCC was constructed based on snRNA-seq, spatial transcriptomics, and pathology WSI data

Given that these cell types co-clustered, including (i) "SPP1_Macro", "Tip-like EC" and "Inflam EC", (ii) "LAMP3_DC3" and "Naive T", (iii) "Plasma B" and "CAF", we named the three paired cell types as an L-TME niche and conducted a co-scoring analysis and classified them as a unique multicellular community in the TLE region of early HCC (Figure 7). After confirming that the L-TME niche could affect the prognosis of HCC, we sought to determine whether the L-TME niche could predict the occurrence of early HCC in patients after surgery. Therefore, to better predict the recurrence of early HCC after surgery, we collected the pathological WSIs of early HCC samples from TCGA and our center, respectively. Further, based on the ResNet50 and CellProfiler, deep learning features and pathological features were successfully captured. Mentioned above, using snRNA-seq and spatial transcriptomics, we deconvoluted the cell types in the leading-edge area of early HCC, and identified an L-TME niche, which might affect HCC prognosis. To explore the potential prediction of early HCC recurrence, we combined the L-TME niche with the pathological features from a pathology database (Figure 8A). Importantly, correlation and Enet analyses were performed for identifying the significantly L-TME niche-related features, leaving 71 deep learning features and pathological features were left, including 65 deep learning features from ResNet-50 and 6 pathological features from CellProfiler (Figure S18). Using a machine learning framework comprising 101 combined algorithms 33, we evaluated a machine learning benchmark for predicting the recurrence of early HCC based on deep learning and pathological features. Intriguingly, we found that the "StepCox[both]+RSF" algorithm could be applied to detect the occurrence of early HCC (Figure 8B-8D). To enhance the interpretability of our machine learning models and identify the most influential features for predicting patient outcomes, we performed SHapley Additive exPlanations (SHAP) analysis. This approach allows the quantification of feature importance at both the global and local levels, providing insights into how each feature contributes to individual predictions. As shown in Figure 8E-8H, the results of the SHAP analysis indicated that the two deep learning features, resnet1064 and resnet1078, contributed the most to the recurrence model. Intriguingly, resnet1064 and resnet1078 played opposite roles in predicting occurrence (Figure 8H), which was in line with the results of unicox analysis (Figure 8D). We then divided patients with early HCC into low- and high-recurrence groups. Of interest, the Gradient-weighted Class Activation Mapping (Grad-CAM) showed that the machine learning model mainly focused on the tumor cells in the low-recurrence group. It also highlighted tumor cells near the vascular area in the high-recurrence group, indicating a potential L-TME niche and vascular metastasis in high-recurrence group (Figure 8I). We validated the model performance using our inhome data, and similar results were obtained (Figure 8J).

Finally, the correlation between the recurrence model and clinicopathological parameters was analyzed. The results revealed that the tumor recurrence and AFP levels were mainly correlated with the model score (Table 1; p = 0.008 and p = 0.012, respectively). Taken together, the L-TME niche, comprising "CAF", "LAMP3_DC3", "Naive T", "Plasma B", "SPP1_Macro", "Inflam EC" and "Tip-like EC", served as a crucial risk factor and predictive indicator for the occurrence of early HCC.

## Discussion

Hepatocellular carcinoma (HCC), as one of the most prevalent malignancies, is the fourth major cause of cancer-associated deaths globally and poses a significant threat to health, particularly due to the high recurrence rate following curative resection, which is often driven by microscopic invasive fronts. 34. The TME of primary tumors is characterized by significant heterogeneity, including both tissue-resident and cells recruited from distant sites. This heterogeneity further escalates as the tumor metastasizes, leading to the formation of spatial heterogeneity. Our study proceeded in three steps: (i) global landscape of cell types in the TLE region (Figure 1), (ii) in-depth sub-clustering of each major lineage (Figure 2-6), and (iii) integrative analysis with pathology to construct a recurrence model (Figure 7-8). This study conducted a comprehensive analysis based on scRNA/snRNA sequencing, spatial transcriptomics and pathomics, comparing the spatial heterogeneity of the invasive front, which aided in understanding tumor behavior, including invasion and metastasis.

The invasive frontier, where tumor cells penetrate the peritumoral tissue and directly interact 8, is acknowledged as the key region for understanding cancer invasion and metastasis 3. Previous reports have indicated that the expression of immune checkpoint genes is enhanced in the TLE region, including CTLA4 in immune cells. Additionally, immune evasion-related features have been found to be upregulated in tumor cells near the boundary line 7. Intriguingly, damaged hepatocytes in the invasive area can secrete SAAs, leading to the specific recruitment of macrophages via "SAA-TLR2" axis 7, which causes local immunosuppression and promotes cancer progression, establishing pro-metastatic niches in the liver 8. In the TLE region of intrahepatic cholangiocarcinoma, another type of liver cancer, cancer cells showed high proliferation and are highly related to stromal cells, including POSTN^+^ FAP^+^ fibroblasts and endothelial cells 35. In this study, we also found that STMN1-high tumor cells were closely associated with immune cells related to immune suppression, such as Naive T cells, SPP1^+^ macrophages, LAMP3^+^ DCs and CAFs in early HCC. Thus, in line with prior research, we highlighted that the more immunosuppressive microenvironment at the invasive front promoted the further invasion and metastasis of malignancies, particularly STMN1-high tumor cells. Although the three STMN1-high subpopulations (C7, C10 and C11) shared high STMN1 expression and poor prognosis, they exhibited subtle transcriptomic differences (Figure 2F). For the purpose of assessing the overall impact of STMN1-high malignant cells on the TLE region and recurrence, we analyzed them collectively. Further studies still need to perform for understanding the differences among the three subpopulations. Moreover, the SCENIC analysis predicted that E2F family members (E2F1, E2F2, E2F7 and E2F8) might regulate STMN1-high tumor cells; however, these were still *in silico* predictions that required experimental validation (ChIP-qPCR or luciferase reporter assays).

Additionally, we found that the TLE region of early HCC had a unique cancer ecosystem. We characterized an enriched tumor cell cluster (STMN1-high tumor cells), which, compared to other regions, exhibited enhanced EMT and activated immune responses. The characterization of this TLE zone provides crucial clinical insights into prognosis prediction and proposes several underlying therapeutic targets for early HCC. STMN1-high tumor cells demonstrated greater proliferative capacity and enhanced interactions with other immune cells and stromal cells compared to STMN1-low tumor cells. These findings underscored the tumor heterogeneity in early HCC, highlighting the necessity for personalized treatment strategies that account for the distinct features of each subtype. We developed a deep learning prediction model indicating that the TLE zone cells were linked to poor prognosis in whole-stage HCC patients. More, the identified spatially resolved L-TME showed strong correlation in terms of infiltration abundance and spatial patterns, collectively promoting immune suppression and tumor progression in early HCC. Particularly, we also compared L-TME scores between patients with early recurrence and those without recurrence. This comparison did not reach statistical significance (data not shown). The lack of a direct association between L-TME score and early recurrence was precisely why we integrated pathological features with the L-TME score to construct a machine-learning recurrence prediction model, which achieved robust performance (C-index = 0.77; log-rank *p* < 0.05 in external validation). With regarding to the relapse of early HCC after surgery, we thereafter combined the snRNA-seq, spatial transcriptomics with pathomics to construct a L-TME-based pathological machine learning model, which could robustly predict the recurrence of early HCC after surgery.

A previous study showed that the mucosal-associated invariant T cells are highly enriched in adjacent tissues and leading-edge area of HCC, contributing to the immunosuppressive tumor microenvironment 36. In this study, we also explored different T cell subsets in early HCC. Our results suggested that STMN1-high tumor cells are closely interacting with Naive T and Treg cells compared to other T cells. More interestingly, we found that LAMP3^+^ DCs exhibit prominent co-localization with Naive T and Treg cells, suggesting that LAMP3^+^ DCs might also influence the recruitment of Naive T and Treg cells. Additionally, we analyzed B cells and observed that plasma B cells show significantly high levels of ERS. Significantly, we observed that CAFs co-localized with plasma B cells in the early HCC leading-edge area (Figure 7A, Figure S9). This indicates that CAFs might contribute to forming a tumor-suppressive TME by suppressing the function of plasma B cells. Using correlation and clustering analyses based on spatial transcriptomics, we identified a close relationship between Tip-like ECs, inflammatory ECs, and SPP1^+^ macrophages, indicating that SPP1^+^ macrophages origined from blood and that Tip-like ECs and inflammatory ECs are closely related to the invasive microenvironment in early HCC (Figure 7A and 7M).

To further characterize the functional states of the identified subpopulations, we conducted computational metabolic flux inference. Although these analyses reflect transcriptional potential rather than direct metabolic measurements, they revealed relative differences that were consistent with the spatial co-localization patterns. Metabolic analysis indicated that the metabolism state was more active in plasma B cells, CAF, naive T cells, LAMP3^+^ DCs and SPP1^+^ macrophages (Figure 3I, Figure S11A-D, Figure S12E-F, Figure 5L and S13D). Cell metabolic analysis also suggested that the metabolism states of inflammatory endothelial cells and Tip-like endothelial cells were more similar with each other (Figure S15D and S15E). Thus, most of the seven cell types, consisting of three co-localized cell types, were in a higher metabolic state than other corresponding sub-cluster cell types, which was also an additional rationale for selecting them to construct the pathological recurrence model.

In this study, we comprehensively characterized the tumor, stromal, and immune cell types in the TLE region of early HCC. We pinpointed distinct gene features and specific spatially resolved cell populations (L-TME) linked to the early HCC boundary region. To validate the robustness of our findings, we employed multiple approaches, including snRNA-seq/scRNA-seq, bulk RNA-seq, spatial transcriptomics, immunofluorescence and digital pathomics. Through the integration of spatial transcriptomics with snRNA-seq/scRNA-seq, we identified the cellular landscape and transcriptomic structure of the TLE region in early HCC patients. Notably, the TLE region was the most active area, characterized by a complex and dynamic TME, with the enrichment of STMN1-high tumor cells and a variety of recruited immune-suppressive immune cells, promoting tumor progression. Our results contributed to the understanding of how the TME influences cancer progression and uncovered active interplay between tumor and immune cells, which might aid in the development of novel therapeutic strategies for early HCC.

However, certain limitations of the present study should be acknowledged. Firstly, the sample size in our work was small, which may constrain the definitive conclusions. To address this problem, we combined publicly available bulk, scRNA-seq and spatial transcriptomics datasets with multiplex immunofluorescence analysis for validation. Nevertheless, additional large-scale studies were required to validate and expand upon our results. Secondly, we constructed a DeepSurv model based on cell composition at the leading-edge area and applied it to public transcriptomic cohorts such as TCGA, which predominantly represented whole-tumor transcriptomes. Although cell types enriched at the TLE region could also be detected over the whole tumor tissue, further model validation in TLE bulk RNA-seq data of large early HCC cohort still needed to strength the biological rationale for its detectability and predictive power at the whole-tumor level. The application of an early-stage-derived cell-type signature to whole-stage HCC was exploratory and also required prospective validation in cohorts with detailed staging and survival data. Thirdly, the inference of multicellular populations from transcriptomics data, requires high-dimensional multiplex *in situ* analysis. Finally, *in vitro* and *in vivo* studies will aid in elucidating the potential molecular mechanisms among the L-TME cell niche.

## Conclusion

In conclusion, our research thoroughly outlines the cellular ecosystem of the leading-edge zone in early HCC. We highlighted that the STMN1-high tumor cells enriched at the TLE region and interacted with Naive T cells and Treg. A L-TME niche was identified by snRNA-seq, spatial transcriptomics and histo-pathomics. A L-TME niche-based machine leanring model could robustly aassess the occurrence of early HCC. This work provides a comprehensive understanding of the heterogeneity present at the tumor invasive front, which might aid in the development of precise and effective therapeutic targets for early HCC.

## Materials and Methods

### Collection of HCC tissue samples

This study was approved by the Research Ethics Committee of the Shanghai East Hospital, Tongji University and the Research Ethics Committee of the Eastern Hepatobiliary Surgery Hospital, Third Affiliated Hospital of Naval Medical University. The inclusion criteria for patients involved the absence of evident metastasis, a single tumor size smaller than (<=) 5 cm, and no previous treatment interventions. Written informed consent was obtained from all participants. Separate surgical resection specimens were collected from early HCC patients. Specifically, surgical samples from early-stage HCC patients who had undergone surgical resection at Eastern Hepatobiliary Surgery Hospital between May 2020 and February 2021 were gathered. The "seven-point baseline sampling" method was employed in this work, with samples taken at a 1:1 ratio from the adjacent and tumor tissue at the the positions of 12, 3, 6, and 9 o'clock. At least one sample was taken from within the tumor, and additional samples were obtained from areas near the tumor margin (≤ 1 cm) and distant from the tumor (> 1 cm), with a total of 7-10 tissue blocks per sample, each approximately "1cm × 1cm × 1cm" in size. After the tissue blocks were obtained, they were rinsed with pre-cooled phosphate-buffered saline (PBS) and dried with lint-free paper. The tissue blocks were then placed in a metal cup containing isopentane that had been pre-frozen with liquid nitrogen for rapid freezing. Afterward, the frozen tissue blocks were placed into 15 ml sterilized centrifuge tubes. The tissue blocks were embedded in optimal cutting temperature compound (OCT) and stored at -80°C. Frozen sections were then stained with hematoxylin and eosin (H&E).

Regarding TCGA samples of early HCC, those patients with entire clinical data, transcriptomic expression data and high-quality WSI pathology data were retrieved and included. Specimens with poor image resolution and missing recurrence information were excluded. Finally, a total of 147 early HCC patients from TCGA were collected and 123 early HCC patients from our center were used in the present work.

### Downstream analysis of scRNA/snRNA-seq in the TLE region of hepatocellular carcinoma

In this study, TLE tissues were obtained from early HCC patients at 0.4-0.6 cm distal to the tumor-adjacent interface on both the tumor and adjacent sides. Specifically, the TLE region was identified by ≥2 experienced liver pathologists on fresh surgical specimens as a strip of tissue 0.4-0.6 cm in width distal to the macroscopic tumor-adjacent tissue interface. This strip was dissected with a scalpel, snap-frozen, and used for snRNA-seq. It was distinct from the tumor core (tissue on the proximal side of the interface) and adjacent non-tumor tissue (beyond 0.6 cm from the interface). The chosen width was consistent with published references (Supplementary Table S1). The next-generation sequencing of the spatial transcriptomics and snRNA-seq was performed on an Illumina NovaSeq 6000 based on the 10× Genomics Visium platform and 10× Genomics platform (Supplementary Table S1). The detailed information of the five patients used for snRNA-seq and the four patients used for spatial transcriptomics was summarized in Supplementary Table S2. The snRNA-seq data (10× Genomics) obtained from TLE tissue samples of HCC were first aligned using Cell Ranger (GRCh38 genome) and gene quantification was performed. Quality control was conducted using Seurat package, keeping high-quality nuclei with gene detection counts ranging from 500 to 6000 and mitochondrial gene proportions < 15%. Cell subpopulations were annotated using classical marker genes: hepatocytes (ALB, APOA2), cholangiocytes (KRT7, KRT19), tumor cells (AFP, GPC3), immune cells (PTPRC), endothelial cells (PECAM1), and fibroblasts (ACTA2, COL1A1). Particularly, for better distinguishing hepatocytes from tumor cells, hepatocytes (benign/non-malignant cells) were also those cells with low CNV scores and tumor cells were cells with high CNV scores. Paired snRNA-seq and spatial transcriptomics data (10× Visium) were integrated to validate the spatial distribution characteristics of specific cell subpopulations in the TLE area. All analyses were performed in R (version: 4.1.1).

### Downstream analysis workflow of spatial transcriptomics sequencing

Spatial transcriptomics data of HCC TLE region tissue were obtained from the 10× Genomics Visium platform, and raw data alignment (GRCh38 reference genome) and gene expression quantification were performed using Space Ranger. Data quality control was performed using Seurat package, retaining all spots. Following normalization, 3000 highly variable genes were chosen for further analysis. GO and KEGG pathway enrichment analyses were conducted on differentially expressed genes using clusterProfiler. H&E-stained images were integrated, and spatial gene expression patterns were visualized utilizing Seurat. All analyses were conducted in R (version: 4.1.1), and key results were visualized using the ggplot2 package.

### Defining the "tumor-to-adjacent" boundary of spatial transcriptomics data

The "tumor-to-adjacent" boundary/interface of the spatial transcriptomics data needed to be clarified for further analysis. Here, we adopted morphological clustering, CNV inference, automated boundary calling, manual polygon gating, signed distance calculation, and gradient visualization to better display the "tumor-to-adjacent" boundary. The specific steps were shown as follows:

(1) Spatial transcriptome data preprocessing and morphological adjustment: Spatial transcriptomics (ST) data from early HCC were processed using the 10× Visium pipeline. Raw count matrices and spatial coordinates were imported into R and a Seurat object was constructed. To incorporate tissue morphology, the raw expression matrix was corrected using the ME_normalize function using stlearn package (https://stlearn.readthedocs.io/en/latest/installation.html), which aslo implemented in the Cottrazm workflow 37. The morphologically adjusted expression matrix was added to the Seurat object as a new assay ("Morph"). This morphology assay was then normalized, variable features were identified, and principal component analysis (PCA) was conducted. Dimensionality reduction (UMAP) and clustering were carried out to obtain morphological clusters.

(2) CNV inference and definition of putative malignant spots: To identify copy number variations (CNV) at spatial transcriptomic data, the infercnvpy package (Python-based, version:0.4.2, https://github.com/icbi-lab/infercnvpy) was used, with the cluster exhibiting the highest expression of lymphocyte signature genes (PTPRC, CD3D, CD79A and so on) as the normal reference. For each spot, the analysis provided a CNV label (CNV_leiden) and a CNV score (CNV_score). The two CNV clusters with the highest CNV scores were selected as putative malignant labels (MalLabel); all spots with the "Normal" CNV label were manually assigned a CNV score of zero to clearly separate them from malignant spots.

(3) Automated boundary definition: The BoundaryDefine function of Cottrazm workflow 37 was then applied. Spots belonging to MalLabel were classified as malignant (Mal). Non-malignant spots that were spatially adjacent to malignant regions and showed intermediate CNV scores were designated as boundary (Bdy) spots. All remaining spots were categorised as non-malignant (nMal), including the normal reference area and other non-malignant tissues.

(4) Manual polygon gating for refined tumor leading edge determination: To further refine the boundary region, interactive manual polygon gating was performed using the GateData::PolygonGating function (https://github.com/xiangmingcai/GateData), which allowed pathologist-guided selection of spots based on spatial coordinates (row and column). A polygon was drawn around the "tumor-to-adjacent" interface to specifically capture spots representing the immediate tumor front. This step integrated algorithmic output (CNV scores and morphological clustering) with expert morphological judgement, resulting in a high-confidence tumor leading edge category, termed TLE_200 μm (tumor leading edge within 200 µm). The final classification thus comprised three categories: "Tumor" (malignant core), "TLE_200 μm" (leading edge), and "Adjacent" (non-malignant). The spot width of 10× Visium is 50-550 μm and the distance from one spot to another spot is 100 μm (https://www.10xgenomics.com/cn/platforms/visium). Thus, the identified boundary spots could be named as "TLE 0-200 μm". It indicated that the boundary spots were involved in the 200 μm width from "tumor-interface-adjacent" (-100, 0, +100 μm) interface.

(5) Spatial distance quantification using POSST: To quantitatively describe the spatial relationship of each spot to the leading edge boundary, the POSST algorithm was adapted (https://github.com/ zaozaozaonan/POSST) 38. For each tissue section, the coordinates of all TLE_200 μm spots were extracted as the boundary reference set. For every spot, the Euclidean distance to the nearest TLE_200 μm spot was calculated. A signed distance (MDB, minimum distance to boundary) was then assigned: negative values for spots located on the tumor core side ("Tumor") and positive values for spots on the adjacent-tissue side ("Adjacent"). TLE_200 μm spots themselves received a distance of zero. The absolute value of MDB represents the spatial displacement from the leading edge. To obtain a discrete, integer-based distance metric (MDBu), the minimum absolute non-zero MDB across all spots was identified as the unit step size. Raw MDB values were divided by this unit step and rounded to the nearest integer. Consequently, each spot acquired a signed integer distance (e.g., -3, -2, -1, 0, 1, 2, 3) that can be directly used for downstream grouping.

(6) Gradient analysis along the leading edge: To validate the biological distinctiveness of the TLE_200 μm region, the bd_line_plot function from POSST package was used. This function plots the expression of feature genes or cell-type enrichment scores against the discretised distance MDBu, spanning from the tumor core, across the leading edge, into the adjacent tissue. This approach quantitatively confirms whether the leading edge functions as a transcriptional transition zone.

### Combination analysis of pathway activation inferred by scRNA-seq data and GWAS summary statistics (ScPagwas) for figuring out pro-tumor-associated cell subpopulations

ScPagwas, uses a multi-gene regression model by integrating pathway activity-transformed scRNA-seq data with GWAS summary data to prioritize trait-related genes and discover trait-related cell subpopulations 16. The present study used snRNA-seq and spatial transcriptomics (ST) data, combined with the scPagwas package (version: 1.3.1), to analyze the molecular characteristics of the TLE region of HCC. Polygenic risk scores (PRS) at the cellular level were calculated based on GWAS-derived liver cancer risk gene sets and spatial mapping was used to analyze high-risk cell clusters in the TLE region. The specific steps included: 1) projecting GWAS signals onto snRNA-seq data using the scPagwas module; 2) identifying regions with significant PRS enrichment in spatial coordinates using the SpaGCN algorithm. Based upon scPagwas, pro-tumor-associated cell subpopulations were successfully identified.

### Cell-cell communication analysis

SnRNA-seq data from the TLE region of HCC were utilized to perform cellular communication network analysis based on the CellChat package. Firstly, the gene expression matrix for each cell subpopulation was extracted using the Seurat package, and a CellChat object was constructed by combining it with the built-in human ligand-receptor database of CellChat. Cell communication analysis using CellChat involved the following core steps: 1) Communication probability calculation: Based on expression abundance and the interaction database, the truncated mean method (trimmed mean = 0.1) was used to estimate communication probabilities between cell populations. 2) Network significance testing: Significant ligand-receptor interactions were identified using permutation testing (100 iterations). 3) Signalling pathway analysis: Multiple ligand-receptor pairs were integrated to calculate the communication strength of each signalling pathway. 4) Network topology features: The flow of information into and out of each cell population was calculated, identifying key signal senders and receivers. The specific interaction patterns were emphasized in the TLE region, with differential comparison to the tumor core region.

### Spatial interaction analysis of tumor leading-edge area based on the mistyR package

The mistyR (Multiview Intercellular SpaTial modeling framework) pipeline, is designed for spatial interaction analysis, primarily used to combine scRNA-seq with spatial transcriptomics data to analyze intercellular spatial interactions and molecular regulatory mechanisms within complex tissue microenvironments 39. This study employed mistyR (v1.4.0) to integrate and analyze snRNA-seq and spatial transcriptomics (10× Visium) data from the TLE region of HCC. Initially, snRNA-seq data were normalized and clustered using Seurat to obtain the characteristic expression profiles of cellular subpopulations. Following Space Ranger processing of the spatial transcriptomics data, SPOTlight was applied for spatial deconvolution to extract cell composition data at each spatial spot. A multi-view analysis framework was constructed using mistyR: 1) "intraview" (spatial autocorrelation model) and "interviews" (microenvironment interaction model) were established, with a spatial neighborhood radius of 100 μm; 2) A multivariate random forest algorithm was used to assess the cell-cell interaction characteristics at the TLE region; 3) Focused on spatial co-localization patterns of cancer cells (STMN1-high), CAFs (FAP^+^) and their molecular regulatory networks. Finally, the spatial dependence index (R²) for each cell subpopulation was computed to identify cell-cell interaction modules that are specific to the TLE region. The results were visualized based on built-in functions of mistyR, and ggplot2 was integrated for data presentation.

### Spatial trajectory inference analysis of the TLE region of HCC (stlearn analysis)

This study employed stlearn (v1.3.1) to analyze the 10× Visium spatial transcriptomics data from the TLE region of HCC (https://stlearn.readthedocs.io/en/latest/installation.html). The following key analyses were performed: 1) Spatial trajectory analysis: pseudotime reconstruction algorithms were used to reveal gene expression gradient patterns from the TLE region to the potential tumor core; 2) Cell neighborhood analysis: a Delaunay triangulation network was constructed based on spatial coordinates to identify cell interaction hotspots specific to the "tumor-non tumor" microenvironment boundary; 3) Spatial differential expression: Moran's I test was used to select genes with significant spatial correlation (FDR < 0.05). The built-in functions of stLearn package were used to visualize spatial gene expression patterns and potential spatial trajectory. All analyses were conducted within the python (version: 3.9) environment.

### Spatial cell-cell interaction network analysis

In this study, the pipeline of COMMunication analysis by Optimal Transport (COMMOT, version: 0.0.3) was utilized to perform cell-cell communication (CCC) analysis on the spatial transcriptomics data (10× Visium) of the TLE region of HCC 40. A custom interaction database containing 896 pairs of high-confidence ligand-receptor interactions was constructed by integrating databases such as CellChatDB and CellPhoneDB, with a focus on signalling pathways related to HCC. The following key analyses were performed using COMMOT python package (version: 0.0.3): 1) Spatial communication scoring: diffusion models calculated the ligand-receptor interaction strength for each spot, with a maximum interaction distance of 200 μm; 2) Network topology analysis: a region-specific interaction network was built for the TLE region, identifying pivotal hub genes; 3) Directionality analysis: spatial gradient algorithms were used to analyze the direction of ligand signal transmission. The results were visualized using built-in functions in COMMOT, including spatial interaction heatmaps, signalling flow direction arrow plots, and other visualizations. Key findings were validated through multiplex immunofluorescence, with co-localization of SPP1^+^ macrophages and STMN1-high tumor cells.

### Calculation of the L-TME score

The L-TME (leading-edge tumor microenvironment) score was a per-sample continuous score that quantified the combined abundance of the three co-localized pairs that constitutes the L-TME niche. These three co-localized pairs, comprsing of seven cell types, were identified from our snRNA-seq and spatial transcriptomics analyses: SPP1⁺ macrophages, LAMP3⁺ DC3, Naive T, plasma B cells, CAFs, inflammatory endothelial cells, and Tip-like endothelial cells. For each sample (from TCGA or our in-house cohorts), we calculated the L-TME score using the following pipeline:

1) BayesPrism deconvolution to obtain cell type abundances: Using our snRNA-seq reference (35 cell subpopulations), we applied BayesPrism to the bulk transcriptomic expression data of each sample. The output was a cell-type-by-sample abundance matrix , where each entry represents the estimated relative abundance of a given cell type in a given sample. The input expression matrix was the normalized count data (FPKM, log₂-transformed) of bulk RNA-seq.

2) Standardization (z-score) across samples for each cell type: To make abundances comparable across cell types (which may have different baseline ranges), we applied z-score standardization to each cell type across all samples: score <- scale(score). After this step, each cell type's values have mean = 0 and standard deviation = 1. This step ensures that cell types with inherently higher abundance do not dominate the final score.

3) Min-max normalization to [0, 1] for each cell type: After scaling, we applied a min-max normalization function to each cell type across samples:



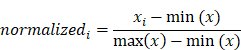



This maps all cell type scores to the same [0, 1] range.

4) Summation to obtain the L-TME score: For each sample, the L-TME score was then calculated as the sum of the normalized scores of all seven cell types in L-TME:



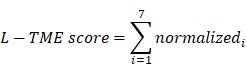



Therefore, each sample received a single numerical L-TME score. Higher scores indicated a greater combined abundance of the three co-localized pairs, named as L-TME cell types, reflecting a more immunosuppressive leading-edge microenvironment.

### Cell type deconvolution analysis using Robust Cell Type Decomposition (RCTD) pipeline

RCTD is a method used to transfer cell type annotations from scRNA-seq data to spatial transcriptomics data. By integrating scRNA-seq/snRNA-seq and spatial transcriptomics data, RCTD enables more accurate assignment of cell types to spatial spots, enhancing the understanding of gene expression in spatial tissue structures 41. This study employed RCTD (Robust Cell Type Decomposition, v2.1.0) for spatial cell type decomposition. First, the snRNA-seq reference data were converted into a Reference object, and high-confidence marker genes (p_val_adj < 0.01, log_2_FC > 0.5) were selected. The "create.RCTD" function was subsequently used to initialized the model, setting "UMI_sensitivity = TRUE" to adjust for sequencing depth differences, and doublet_mode = "multi" was applied to identify spots with mixed cell types. After running "run.RCTD" function, the cell type proportion matrix for each spot was extracted, and results with a confidence score (weights) > 0.8 were retained for subsequent analysis.

### Survival analysis for TCGA HCC patients

This research obtained RNA-seq transcriptomic data (FPKM values) and corresponding clinical information for patients with liver hepatocellular carcinoma (LIHC) from the TCGA database (https://portal.gdc.cancer.gov/). A total of 50 non-tumor samples and 374 HCC samples were included. Data preprocessing was conducted using R (version 4.1.1): 1) Batch correction was performed using the limma package; 2) FPKM values were converted to TPM values and subjected to log2(TPM+1) transformation using edgeR. Survival analysis was performed utilizing the survival (version: 3.4.0) and survminer (version: 0.4.9) packages: 1) Key gene (STMN1) was grouped by median expression levels, and Kaplan-Meier survival curves were plotted; 2) Key cell subgroup (STMN1-high tumor cells) was grouped by median expression levels, and KM analyses were generated. All statistical analyses were performed in R.

### Spatial neighborhood analysis of cell types in TLE region

The spatial connectivity between different cell types, reflecting the strength of their colocalization, is often strongly associated with disease prognosis 42. This research leveraged spatial transcriptomics to primarily examine the spatial colocalization patterns of cell types in the TLE region of HCC. After preprocessing the tissue samples, we perform spatial proximity analysis using the Cell Degree method 42. Initially, cells were annotated based on morphological features and gene expression profiles, followed by the construction of a cell adjacency network with an interaction distance threshold set at 50 μm to reflect the local microenvironment structure. By calculating the colocalization index (CI) between cell types, the aggregation tendency and spatial dependency of various cell types were evaluated in the TLE region. Statistical testing was performed using permutation tests (1000 repetitions) to assess the significance of the colocalization pattern (p-value < 0.05 was considered significant).

### Intercellular interaction analysis using spatial cellular estimator for tumors (SpaCET)

SpaCET (R Version: 1.0.0) was used for studying spatial transcriptomics to investigate cellular interactions in the TME 23. In brief, SpaCET initially estimated tumor cell abundance by integrating gene expression patterns from common malignancies. Moreover, SpaCET integrated scRNA-seq datasets as custom references for cell type deconvolution, analyzing the spatial colocalization patterns of cell types in the TLE region of HCC. The analysis procedure was as follows: first, the preprocessed transcriptomic and spatial coordinate data were imported into SpaCET pipeline, where the built-in function "estimateCellTypeAbundance" was used to infer the cell type proportions at spatial resolution. Next, the "calculateColocalization" function computed the colocalization index for pairs of cell types in the designated TLE region. This index, based on the spatial distance matrix and cell type abundance data, applied a linear regression model to evaluate the spatial association of cell types in TME. To quantify the statistical significance of colocalization, a permutation test (1000 iterations) was used to estimate a null distribution and calculate the empirical p-value (p < 0.05 was considered significant). Lastly, the "visualizeColocalization" function was used to generated spatial colocalization heatmaps and network plots, visually displaying significantly colocalized cell type pairs in the TLE area of early HCC.

### A deep learning prognostic model constructed based on the cellular types in the TLE region of HCC

The DeepSurv package (version 0.4.1) was applied to construct a survival analysis model based upon cellular type composition features, in order to investigate the correlation between the spatial distribution of cells in the TLE area of HCC and patient prognosis 15. The following analysis steps were performed: first, the absolute abundance of each cell type (a total of 35 celltypes) in the TLE region, was treated as a key covariate and integrated with the corresponding clinical survival time and outcome event data. The "deepsurv" function in the DeepSurv package was then used to build a deep survival analysis neural network model. The model learned the intricate relationship between cellular composition and survival risk via multiple layers of nonlinear transformations. To evaluate model performance, the concordance index (C-index) was estimated using bootstrap resampling (500 iterations) to quantify the predictive discriminative ability of the trained model. In the final step, individual survival predictions were output using the "predict_survival" function, and visualization was carried out using "plot_survival_curves". All analyses were performed in a Python 3.9 environment.

### Pathological features extraction

All whole-slide images (WSI) from patients with early HCC were obtained from the TCGA database and our hospital's database, which were stained with H&E and then scanned at magnifications of 20× and 40×. To ensure image quality and consistency, the WSIs were processed using a standardized procedure. All WSIs were segmented into non-overlapping tiles of 512×512 pixels. Initially, the deep learning model, ResNet-50, was employed as the feature extraction model due to its strong performance in convolutional neural networks (CNNs), especially for image classification and feature extraction. The deep residual structure of ResNet-50 allowed for the effective extraction of deep multi-scale features from the input early HCC tissue images. A pre-trained ResNet-50 model was fine-tuned using transfer learning to adapt to our early HCC datasets. In particular, the OpenCV function was applied to process the pathology images of early HCC. To confirm color consistency and remove format-associated discrepancies, the cv2.cvtColor() function was employed to convert all tiles to RGB (red, green and blue) color space. Normalization was then carried out using the ImageNet standard. The tiles were resized to 224×224 pixels. Feature extraction was performed based on the pre-trained ResNet-50 model from the PyTorch. Before the final classification layer, the output of layer 4 was retained, which generated a deep feature map (2048×7×7 size).

Next, CellProfiler (version:4.2.8), a powerful biological image analysis tool, was used to automatically extract various cellular morphological features and conduct quantitative image analysis through algorithms. A specific analysis pipeline was set up in CellProfiler to identify cellular regions in liver cancer tissue and extract relevant morphological features (such as cell size, shape, and texture). These features contributed to uncovering the microstructural traits of the TLE area of early HCC. In the study, H&E stained images were processed utilizing the "UnmixColors" module in CellProfiler to convert the RGB image into grayscale color-separated channels. Optical density deconvolution was used to effectively identify the hematoxylin and eosin components. Based on the segmented nuclei, the "IdentifySecondaryObjects" module was used to delineate the cytoplasmic region, with the module propagating outward from the nuclear boundary to figure out the entire cell region. After segmentation, quantitative features was extracted from all identified objects. These comprised intensity-related features, texture features, shape features, and pixel distribution features. In terms of multiscale feature analysis, deep features extracted by ResNet-50 were able to capture advanced structural information in liver cancer images, while CellProfiler focused on cellular-level morphological changes. By combining the results of both analyses, a deeper understanding of the pathological characteristics of the TLE region of early HCC was achieved at multiple scales. Lastly, the features extracted by ResNet-50 and CellProfiler were combined, and statistical learning methods (such as random forests, support vector machines (SVM), etc.) were used for classification and evaluation of the early HCC TLE region, for providing a more precise recurrence model for early HCC in clinical settings.

### A machine learning benchmark used for early recurrence prediction of early HCC

In order to evaluate the potential of pathological features extracted by CellProfiler software (version:4.2.8) and ResNet-50 model in predicting recurrence of early HCC, a machine learning framework was implemented using the Mime1 R package 33. Initially, univariate Cox regression analysis was performed to assess the relationship between each pathological feature and recurrence within the early HCC cohort from TCGA database. The TCGA-early HCC cohort (n = 147) was stochastically regrouped into a training dataset (n = 104, 70%) and an validation dataset (n = 43, 30%) using a fixed random seed (set.seed(666)). The independent in-house cohort (n = 123) served as the external testing dataset. Feature selection was conducted on the training set in two stages: (i) correlation filtering with the L-TME score (Spearman |ρ| > 0.2), followed by (ii) univariate Cox regression (p < 0.05) and Elastic Net regularization (alpha = 0.5, 10-fold CV). This yielded 71 features for benchmarking.

A total of 101 mechine learning combinations by several machine learning algorithms, such as LASSO, Elastic Net (Enet), CoxBoost, and survival partial least squares regression (plsRcox), were benchmarked for the establishment of the prognostic model using Mime1 pipeline 33. The model performance was estimated according to the concordance index (C-index). Additionally, risk scores were calculated and patients were classified into high- and low-risk groups according to the median risk score. KM analysis was conducted to evaluate the recurrence outcomes between different groups. After correlation filtering (L-TME score correlation), univariate Cox regression (p < 0.05), and Elastic Net regularization, we retained 71 features (65 ResNet-50 features + 6 CellProfiler features) as candidates. These 71 candidate features were then subjected to bidirectional stepwise Cox regression (StepCox[both]) using AIC as the entry/exit criterion. This process selected a minimal optimal feature subset based on predictive power and parsimony. StepCox[both] retained exactly 5 features: resnet1064, resnet1078, resnet618, resnet573, and resnet127. These 5 features are the only features used as inputs to the RSF model. No additional manual feature filtering was applied after StepCox[both].

### SHAP analysis for model interpretation

The SHapley Additive exPlanations (SHAP) analysis was conducted using the fastshap package (Version: 0.1.1) with complementary visualization through shapviz (Version: 0.10.1) and kernelshap (Version: 0.8.0) packages. We focused on the best-performing model identified through our previous machine learning pipeline. SHAP analysis was performed on the "StepCox[both]+Random Survival Forest (RSF)" model, which used the five selected features (resnet1064, resnet1078, resnet618, resnet573 and resnet127) as inputs. Mean absolute SHAP values were calculated to rank the contribution of each of these 5 features to the model's predictions.

For SHAP value computation, we defined a custom prediction wrapper function tailored to the RSF model structure. The function was designed to extract risk scores using the `predict()` method with `predicted` output. We set the random seed to 666 for reproducibility and performed the analysis using the training dataset (Pathology_TCGA) containing five key resnet features (resnet127, resnet573, resnet618, resnet1064, and resnet1078). The resulting SHAP values were processed to remove non-informative features (those with zero contribution) and subsequently visualized through multiple complementary approaches. We generated: (1) bar plots displaying feature importance relied on values of the mean absolute SHAP; (2) bee swarm plots illustrating the distribution of feature effects; and (3) force plots demonstrating individual prediction explanations.

### Immunohistochemical (IHC) analysis of the TLE region in HCC

Formalin-fixed, paraffin-embedded (FFPE) liver samples from patients with early HCC were acquired from the Eastern Hepatobiliary Surgery Hospital. For each case, tissue sections (4-μm thick) containing both the tumor core and the TLE region were specifically selected. The staining was performed following a standardized protocol. Briefly, sections of liver samples were deparaffinized in xylene and then rehydrated using a graded ethanol series. Heat-induced epitope retrieval was conducted using citrate buffer in a pressurized decloaking chamber at 121°C for 2 minutes. Then, they were incubated with 3% hydrogen peroxide at room temperature for about 15 minutes. Sections were then incubated overnight at 4°C with primary antibodies. IHC staining in the predefined TLE region was evaluated semi-quantitatively based on both the staining intensity and the percentage of positive tumor or stromal cells. MVI area was identified independently by two experienced pathologists who were blinded to the clinical information. Primary antibodies adopted in the IHC assay were displayed in Supplementary Table 3.

### Hematoxylin and Eosin (H&E) staining

Routine H&E staining was applied to stain all early-stage HCC tissue samples (a total of 123 cases) involved in the study, in order to assess basic histopathological features and accurately define the TLE region of early HCC. First, liver sections were placed in a 65°C constant-temperature oven for 2 hours of baking, followed by dewaxing with xylene I and xylene II for 10 minutes each. They were then hydrated sequentially with ethanol gradients. They were stained with Hematoxylin for 5 minutes, washed with tap water to remove excess color, then differentiated with 1% hydrochloric acid ethanol solution for 3 seconds, followed by blueing with tap water for 10 minutes. The sections were subsequently stained with a 0.5% Eosin solution for 2 minutes. After staining, the sections were dehydrated through 75%, 85%, 95%, and 100% ethanol gradients, each for 2 minutes, and then cleared with xylene I and II for 5 minutes each. Finally, these sections were mounted with neutral resin. All HE-stained slides were independently examined in a double-blind setting by two hepatobiliary pathologists with more than 10 years of experience. The evaluation focused on tumor differentiation, microvascular invasion, satellite nodules, and others.

### Immunofluorescence analysis

Immunofluorescence double-labeling technology was used in this study to perform co-localization analysis of multiple cell types in the TLE region. HCC tissue samples were frozen and sectioned (6 μm), fixed with 4% paraformaldehyde, and then incubated with primary antibodies specific to the study. Subsequently, secondary antibodies were incubated in the dark for 1 hour, and DAPI was used for nuclear counterstaining. Co-localization analysis was quantitatively performed and statistically analyzed using ImageJ. Statistical tests were conducted using GraphPad Prism software. Negative controls (PBS replacing the primary antibody) were set in all experiments to validate the staining specificity. Primary antibodies utilized in the immunofluorescence assay were shown in Supplementary Table 3.

### Statistical analysis

The one-way analysis of variance was adopted for comparisons involving multiple groups and the Wilcoxon rank-sum test was conducted for the comparison of continuous variables (gene expression and pathway scores) between two groups. The "survdiff()" function was used to classify patients in the HCC cohort. Kaplan-Meier survival curves were applied to assess cumulative survival times, and the log-rank test was used to evaluate survival differences. Statistical significance was indicated as p < 0.05, p < 0.01, p < 0.001, and p < 0.0001, represented by *, **, ***, and ****, respectively.

## Supplementary Material

Supplementary figures and tables.

## Figures and Tables

**Figure 1:**
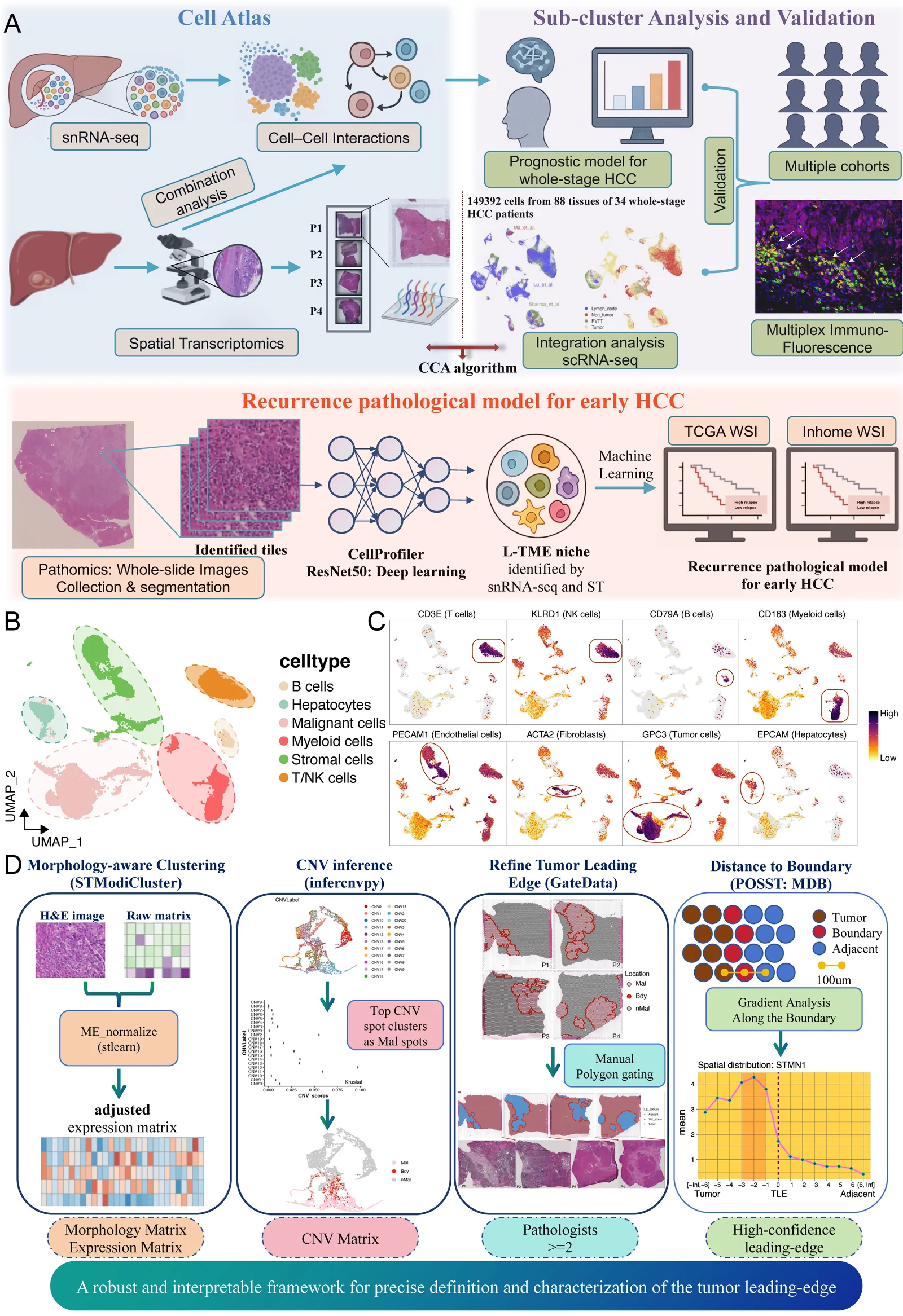
** Spatial landscape of leading-edge region of early HCC.** (A) Flowchart of the present study. Three major aspects of the study were shown: "Cell atlas of early HCC", "Subcluster analysis of each cell types" and "Establishment of relapse pathological model for early HCC". (B) UMAP plot of all detected cell types from early HCC leading-edge tissues based on snRNA-seq. (C) Gene expression of canonical markers for all cell types. (D) A robust framework for precise definition of the TLE region for early HCC. Combination analysis of morphology data, expression data and CNV data were performed.

**Figure 2:**
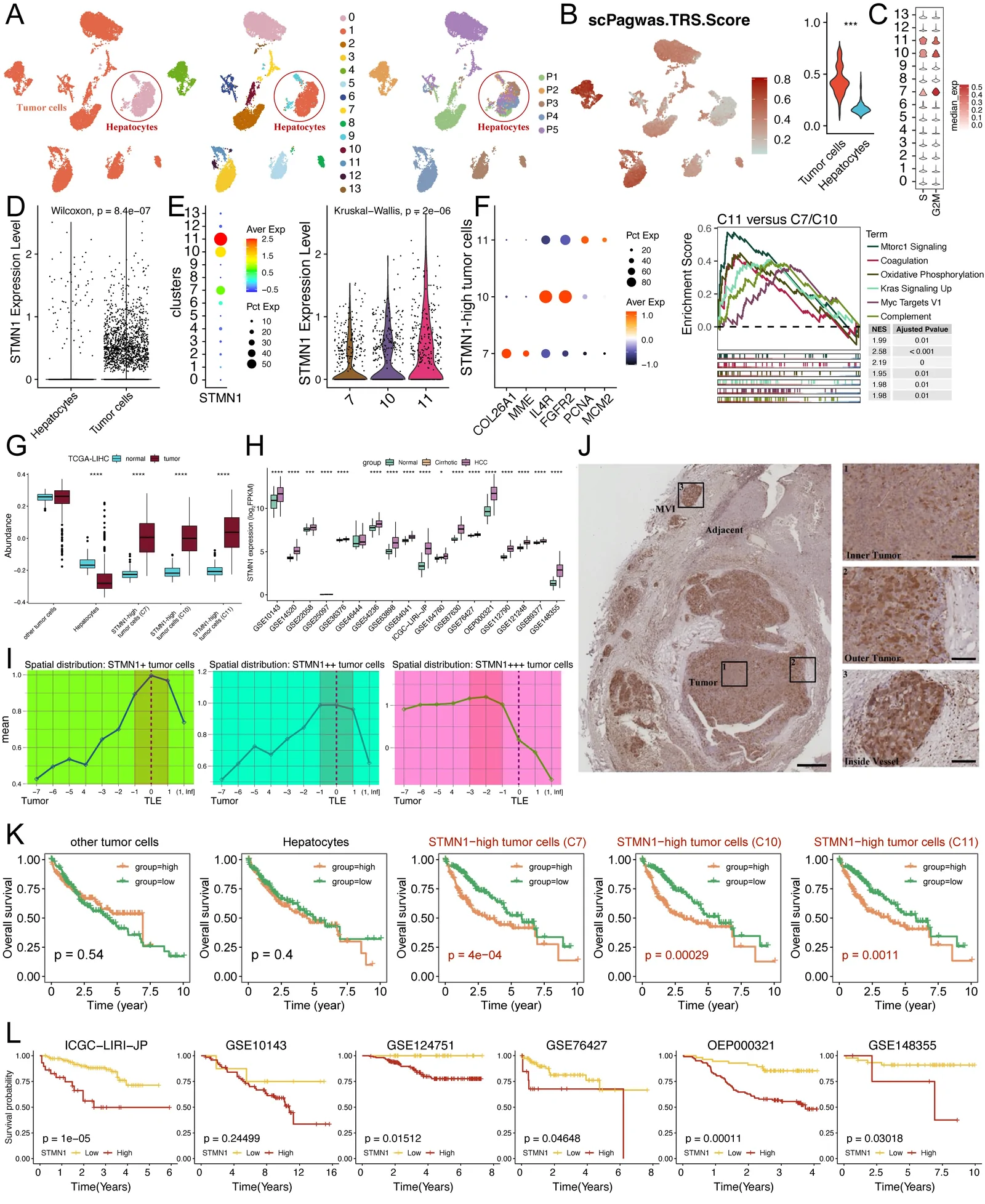
** SnRNA-seq analysis of parenchymal cells of early hepatocellular carcinoma (HCC), highlighting the crucial prognostic role of STMN1-high tumor cells.** (A) Sub-population analysis of parenchymal cells, including tumor cells and hepatocytes. (B) scPagwas score for all tumor cells and hepatocytes. (C) Cell cycle score for all tumor cell clusters. S stage and G2M stage of Cell cycle were calculated using Seurat package. (D) Comparison of STMN1 expression between tumor cells and hepatocytes (average expression of single cells). (E) Dotplot shows the expression levels of STMN1 in tumor cell clusters. Result indicated that the expression levels of STMN1 in Cluster 7, 10 and 11 were much higher than other cell clusters. (F) Several differentially expression genes among the three STMN1-high tumor cells (Cluster 7, 10 and 11). The expression of MME and COL26A1 were higher Cluster 7; The expression of IL4R and FGFR 2 were higher Cluster 10; The expression of MCM2 and PCNA were higher Cluster 11. GSEA results showed that Cluster 11 had higher proliferative and tumor potential than Cluster 7 and 10. (G) The abundance comparisons between tumor and non-tumor tissues of these STMN1-high tumor cells (Cluster 7, 10 and 11), other tumor cells and hepatocytes in TCGA-LIHC database. (H) Expression levels of STMN1 between tumor and non-tumor tissues in more than 15 whole-stage HCC datasets, related to Figure S6B. (I) Dynamic changes of STMN1-high tumor cells in different regions in TLE. Scheme of boundary and distance to boundary analysis methods were performed using the POSST pipeline (position analysis of sequencing-based spatial transcriptomics, POSST, https://github.com/ zaozaozaonan/POSST). The boundary spots were identified using flowchart in Figure 1D. Each layer was about 100 μm in width. (J) Immunohistochemical staining of STMN1 expression. (K) Survival analysis of these STMN1-high tumor cells, other tumor cells and hepatocytes in TCGA-LIHC database. The three survival curves appear similar because the high-/low-risk group assignments based on the three STMN1-high subpopulations (C7, C10 and C11) were highly overlapping due to their correlated abundances. (L) Survival analysis of STMN1 expression in another six datasets with survival information, related to Figure S6C.

**Figure 3:**
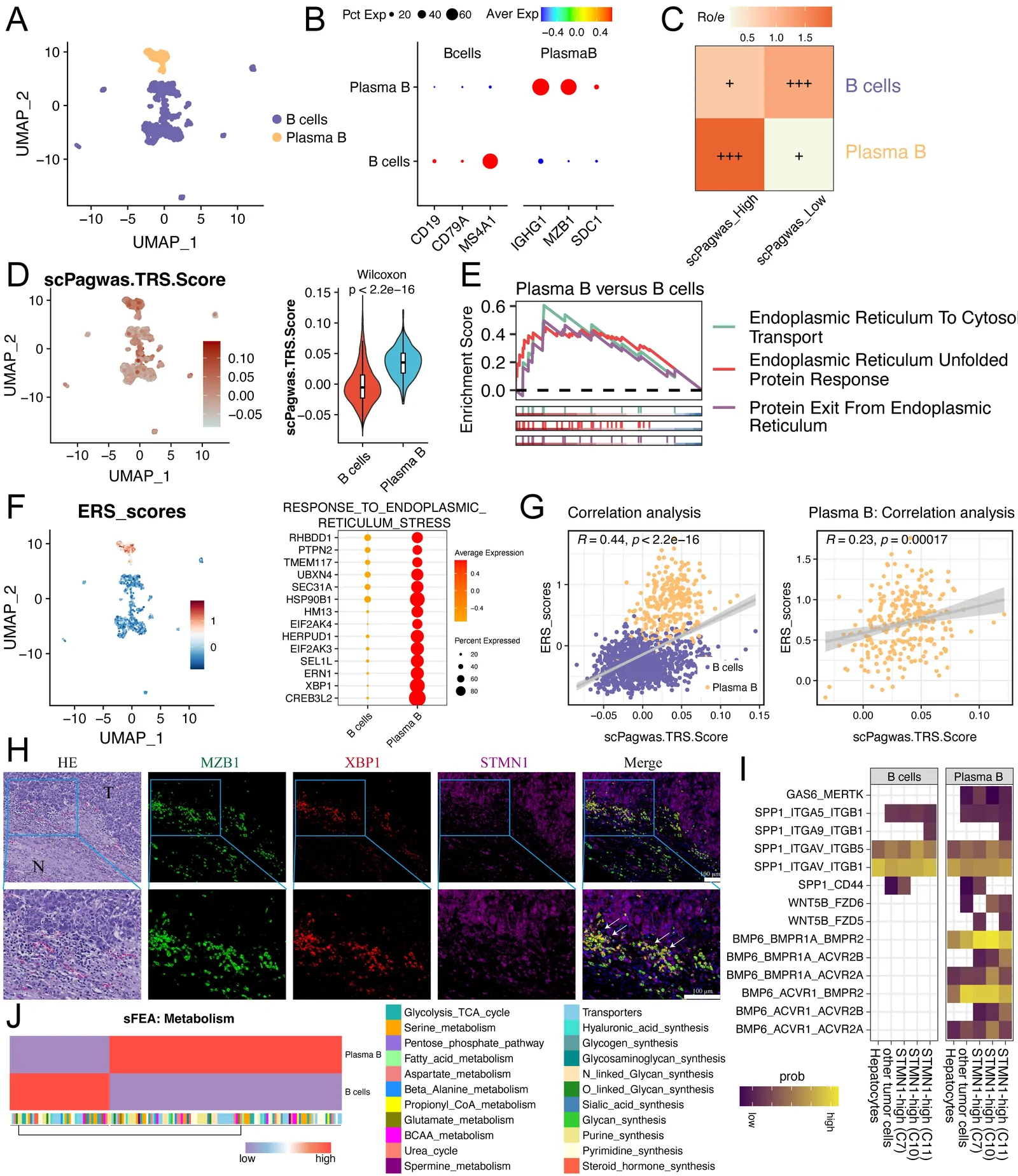
** SnRNA-seq analysis of B cells and plasma B cells.** (A) UMAP plot displays the cell distribution of B cells and plasma B cells. (B) Biomarkers of B cells and plasma B cells. (C) Ratio of observed versus expected cell numbers in scPagwas score-high and scPagwas score-low group. Ro/e: the ratio of observed over expected cell numbers. (D) scPagwas score of B cells and plasma B cells. (E) GSEA algorithm for inferring the enriched pathways in plasma B cells. Only ERS-related pathways were shown. (F) ERS score of B cells and plasma B cells. Dot plot shows the expression levels of ERS-related genes between B cells and plasma B cells. (G) Immunofluorescence assays for plasma B cells, ERS biomarker (XBP1) and STMN1-high tumor cells. (I) Cell-cell interactions among B cells, plasma B cells and STMN1-high tumor cells. Plasma B cells had higher potentials for interacting with STMN1-high tumor cells. (J) Heatmap shows the metabolic flux among plasma B cells and B cells. Most metabolic fluxes in plasma B cells and B cells were quite distinct.

**Figure 4:**
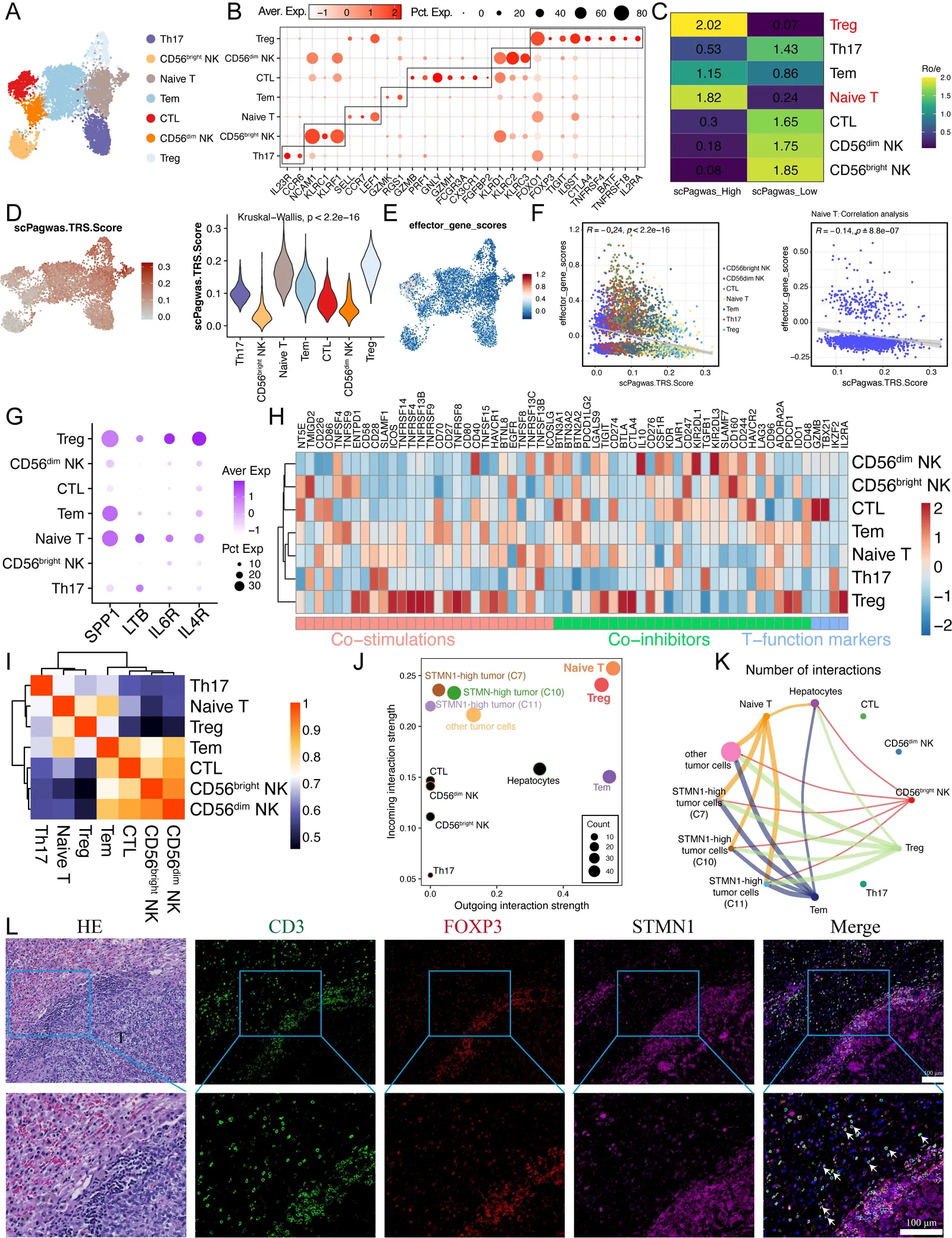
** SnRNA-seq analysis of T/NK cells.** (A) UMAP plot displays the cell distribution of T and NK cells. (B) Expression levels of Biomarkers of T/NK cells. (C) Ratio of observed versus expected cell numbers in scPagwas score-high and scPagwas score-low group. Ro/e: the ratio of observed over expected cell numbers. (D) scPagwas score of T cells and NK cells. (E) Score of T effector-related genes estimated by AddModule algorithm. (F) Correlation analysis of scPagwas score and T effector score in all T/NK cells, and Naive T cells, respectively. (G) Expression levels of SPP1, LTB, IL6R and IL4R in all T/NK cells. (H) Expression levels of genes related to co-stimulators, co-inhibitors and T-function markers in all T/NK cells. (I) Clustering analysis of all T/NK cells. Treg and Naive T cells were clustered together. (J) Scatter plot displays the incoming and outgoing interaction strength based on cellchat pipeline. (K) Number of interactions among all T/NK cells and all parenchymal cells. (L) Immunofluorescence of Treg (FOXP3) and STMN1-high tumor cells. The detailed multiplex immunofluorescence staining results of DAPI and FOXP3 were shown in Figure S10.

**Figure 5:**
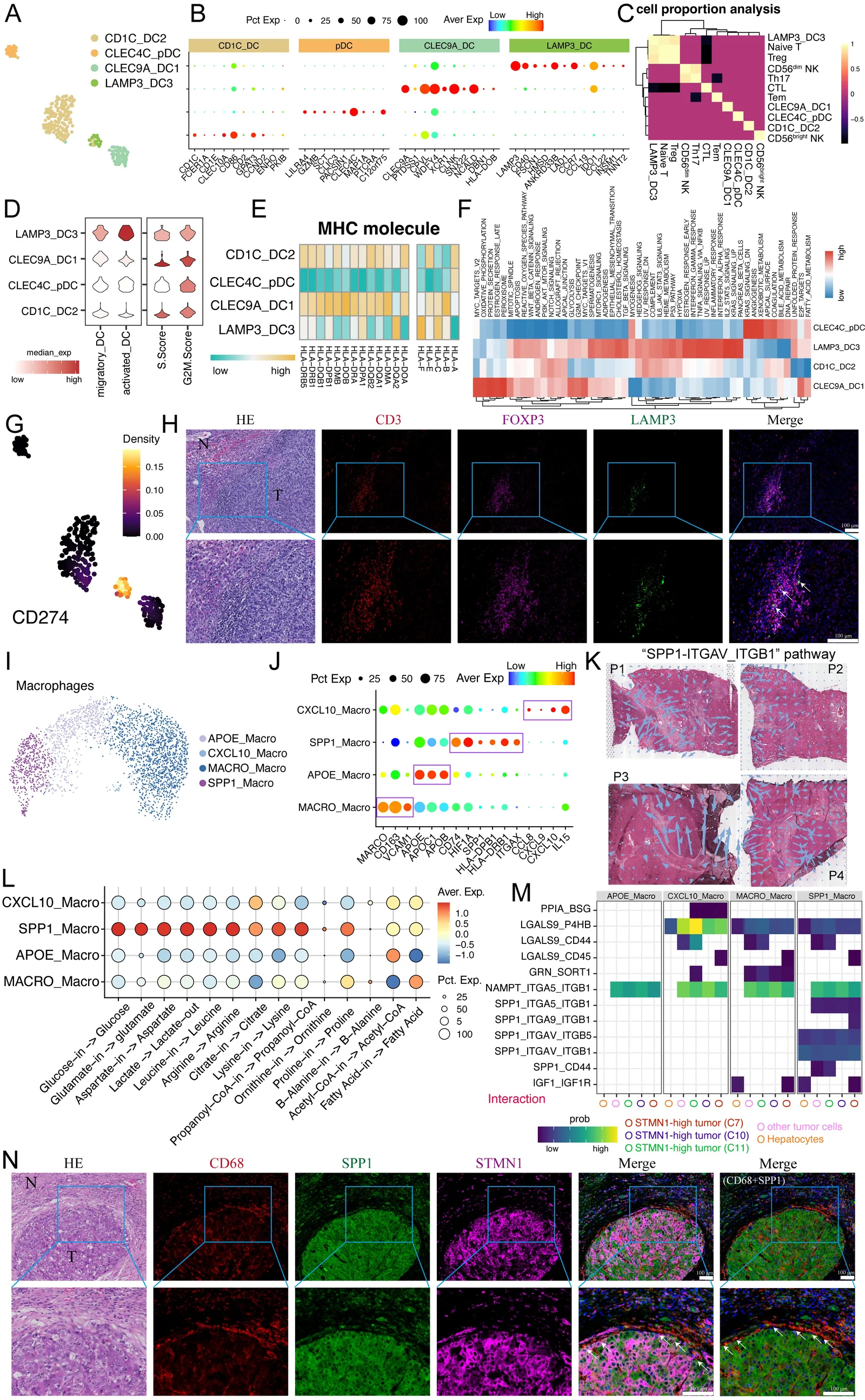
** SnRNA-seq analysis of myeloid cells.** (A) UMAP plot displays the cell distribution of DCs. (B) Expression levels of biomarkers of DCs. (C) Cell proportion correlation analysis of DCs and T/NK cells. The cell proportion of LAMP3^+^ DCs were highly correlated with that of Treg and Naive cells. (D) Functional scores of DCs. Pathway scores, including migratory, activated, cell-cycle-related pathways, of the four identified DCs based on AddModule algorithm. (E) Expression levels of genes related to MHC families. (F) Pathway scores of the four identified DCs based on GSVA algorithm. (G) CD274 expression levels in all DCs. (H) Immunofluorescence assays for Treg (FOXP3) and LAMP3^+^ DCs. (I) UMAP plot displays the cell distribution of macrophages. (J) Expression levels of Biomarkers of macrophages. (K) "SPP1-ITGAV_ITGB1" pathway score in spatial transcriptomic data calculated by the COMMOT package in python. (L) Metabolic flux of glycolysis TCA cycle metabolism in macrophages. The metabolism flux of glycolysis TCA cycle in SPP1^+^ macrophages was highest. (M) Cell-cell interaction analysis between macrophages and tumor cells. (N) Immunofluorescence assays for SPP1 macrophages and STMN1-high tumor cells.

**Figure 6:**
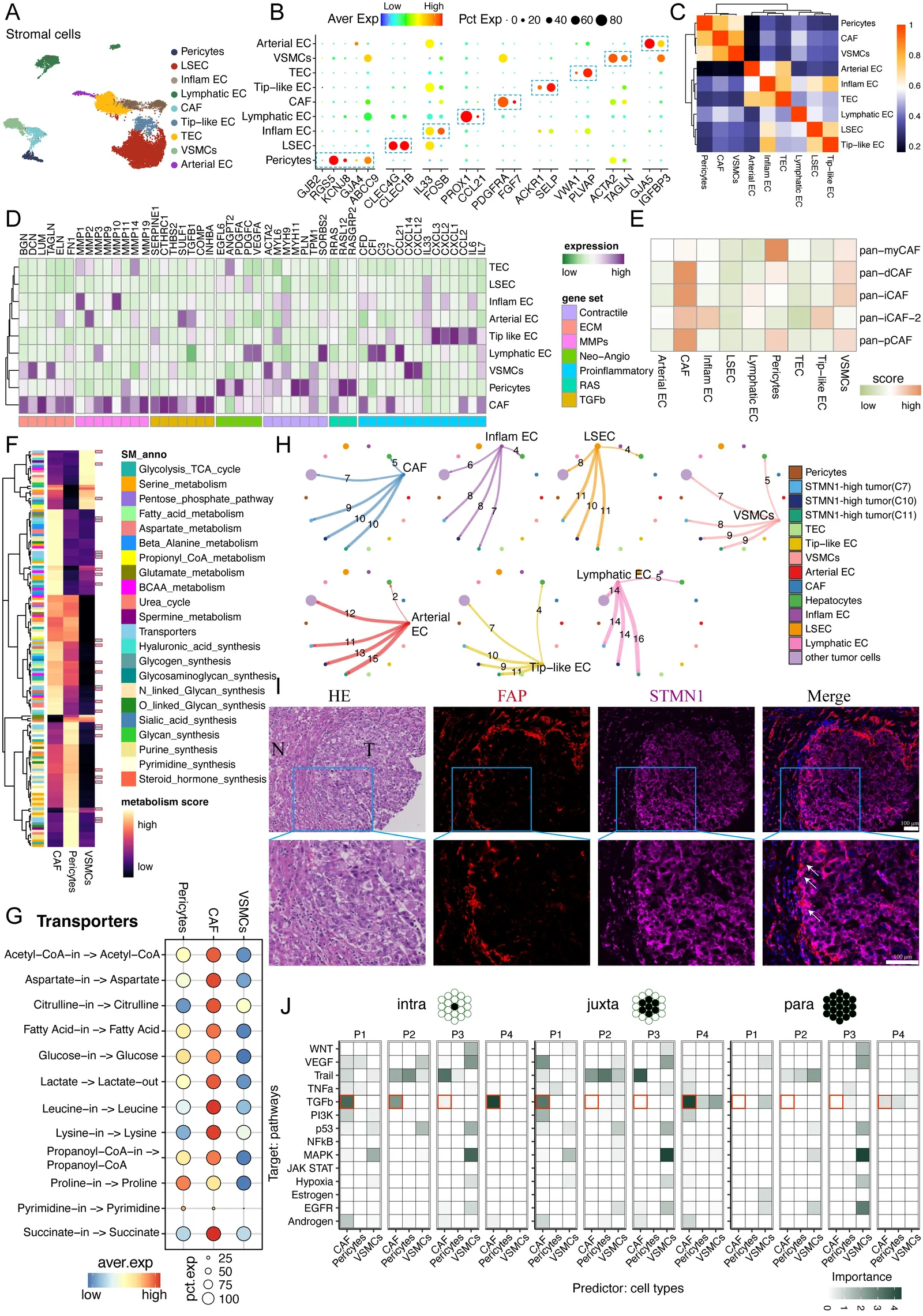
** SnRNA-seq analysis of stromal cells.** (A) UMAP plot displays the cell distribution of stromal cells. (B) Expression levels of Biomarkers of stromal cells. (C) Correlation analysis among stromal cells based on expression profiles. (D) Expression levels of genes related to functions of stromal cells, including contracts, ECM, MMPs, neo-angiogenesis, pro-inflammatory, RAS and TGFB terms. (E) Gene set scores of several identified pan-cancer CAF sub-clusters. CAF had exerted most of these CAF characteristics. (F) Heatmap shows the metabolic flux among identified mesenchymal cells, involving CAF, pericytes and VSMCs. Most metabolic fluxes in mesenchymal cells were quite distinct. (G) Metabolic flux of transports metabolism in mesenchymal cells. The metabolism flux of transports in CAF was highest compared to the other two mesenchymal cells. (H) Cell-cell interaction analysis between stromal cells and tumor cells. (I) Immunofluorescence assays for CAF and STMN1-high tumor cells. (J) Activities of crucial pathways in mesenchymal cells based on mistyR and Progeny package.

**Figure 7:**
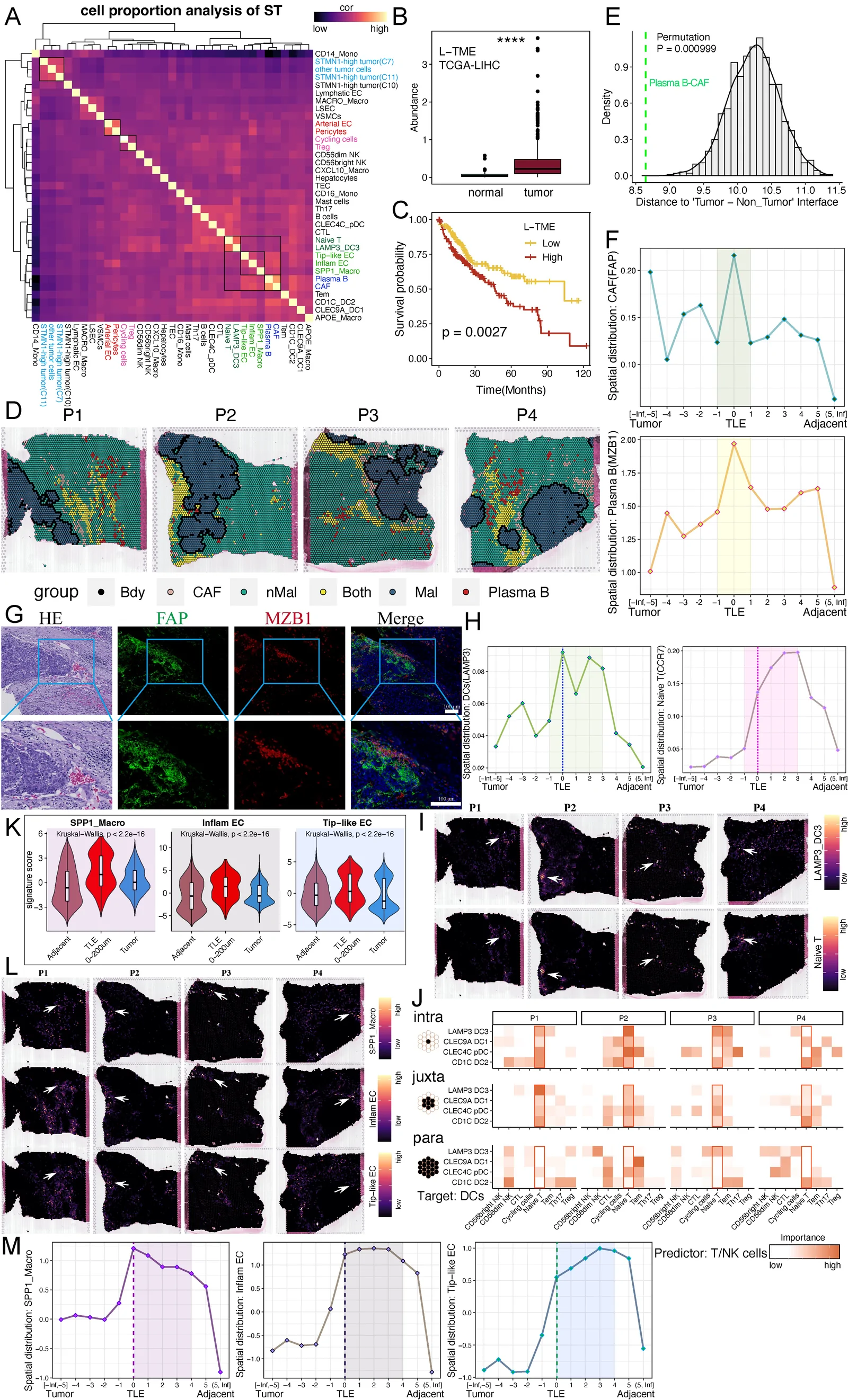
** Characterization of the leading-edge cell niche at the TLE region of early HCC.** (A) Cell proportion analysis of spatial transcriptomic data. (B) Cell abundance of the identified leading-edge TME (L-TME), comprising "CAF", "Inflam EC", "LAMP3_DC3", "Naive T", "Plasma B", "SPP1_Macro" and "Tip-like EC", in LIHC between normal and tumor tissues. (C) Survival analysis of leading-edge TME in LIHC datasets. (D) The distribution of identified CAF spots, plasma B spots and CAF-plasma B (both) spots. The predicted boundary spots were identified and used here using Cottrazm workflow without manual selection, for avoiding potential preference. Bdy: Boundary. (E) Distance to "Tumor-Nontumor" interface of CAF spots, plasma B spots and CAF-plasma B (both) spots. (F) Dynamic changes of CAF and plasma B in different regions in TLE. Scheme of boundary and distance to boundary analysis methods were performed using the POSST pipeline (https://github.com/zaozaozaonan/POSST). The high-confidence boundary spots were identified using flowchart in Figure 1D. Each layer was about 100 μm in width. (G) Co-localization of CAF and plasma B using the IF assay. (H) Dynamic changes of LAMP3_DC3 and Naive T in different regions in TLE. (I) Spatial distribution of LAMP3^+^ dendritic cells and Naive T cells. (J) Cell-cell co-localization analysis of dendritic cells with T/NK cells. The Naive T cells were highlighted. The cell-cell co-localization analysis was performed using mistyR package. (K) Violin plot shows the signature score of SPP1^+^ macrophages, inflammatory endothelial cells and Tip-like endothelial cells at different area of early HCC. The scheme of identifying boundary spots (TLE, 0-200 μm) was shown in Figure 1D. (L) Spatial distribution of SPP1^+^ macrophages, inflammatory endothelial cells and Tip-like endothelial cells. (M) Dynamic changes of SPP1^+^ macrophages, inflammatory endothelial cells and Tip-like endothelial cells in different regions in TLE.

**Figure 8:**
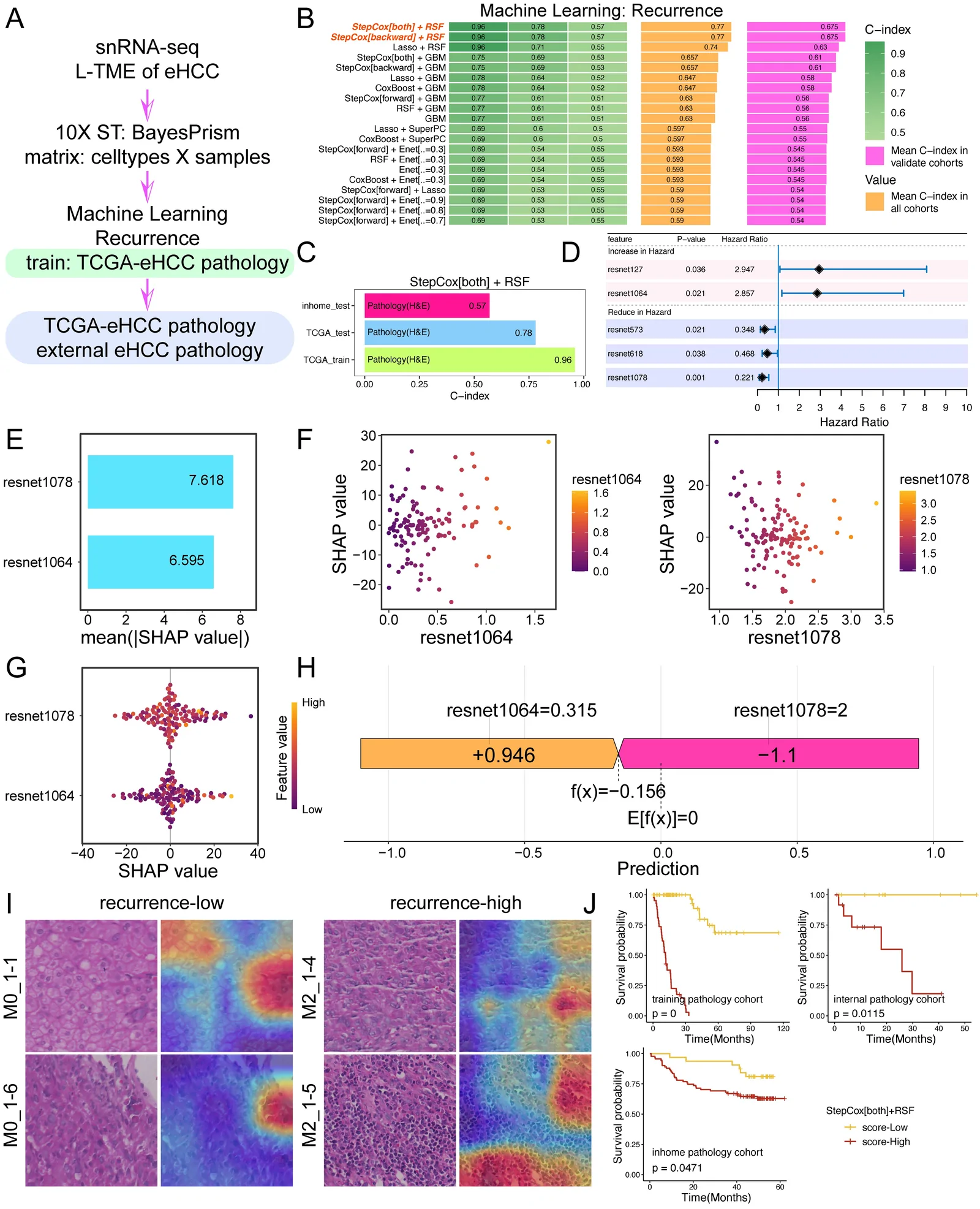
** Construction of a recurrence model based on pathological features and deep learning features.** (A) Flowchart of construction of a L-TME-related recurrence prediction model for early HCC. (B) A total of 101 kinds of machine learning prediction models based on the Machine learning-based integration model with elegant performance (Mime) R package 33 and further calculated the C-index of each model of the internal and external testing early HCC datasets. Only top 20 combined algorithms were shown. (C) C-index value of the "StepCox[both]+RSF" algorithm in the training, internal and external testing datasets. (D) Forest plot shows that hazard ratio of the pathological features selected by the univariate cox algorithm. (E) SHAP analysis for explaining the recurrence model of early HCC. Bar plot shows the SHAP value of crucial deep learning features. (F) Bee plot displays the distribution of SHAP value of crucial deep learning features. (G) Relationship between SHAP value and the scores of crucial deep learning features. (H) Force plot shows the prediction value of crucial deep learning features. (I) Grad-CAM (Gradient-weighted Class Activation Mapping) plots the potential interested area captured by the "StepCox[both]+RSF" algorithm. (J) Recurrence analysis of the L-TME based pathological machine learning model for early HCC.

**Table 1:** Correlations between relapse riskscore calculated by the "StepCox[both]+RSF" machine learning model and clinicopathologic features in early HCC patients.

Features	Total	Low-relapse riskscore	High-relapse riskscore	*p* value
Sex				0.894189
Male	97	26	71	
Female	26	6	20	
Age				0.094674
< 60	82	17	65	
≥ 60	41	15	26	
HBsAg				0.801059
Negative	19	5	14	
Positive	104	27	77	
Serum AFP				0.012639
< 400	84	28	56	
≥ 400	39	4	35	
Tumor size (cm)				0.063819
< 2	23	10	13	
≥ 2	100	22	78	
Cirrhosis				0.981923
Absent	79	21	58	
Present	44	11	33	
Vascular invasion				0.153314
Absent	43	15	28	
Present	80	17	63	
Recurrence/metastasis				0.008058
Absent	87	29	58	
Present	36	3	33	

## Data Availability

The used datasets of whole-stage HCC can be collected in online repositories. All analyses in the present work were conducted in Python and R using standard protocols from previously published packages. The used data (accession number BioProject ID: PRJCA035412) in the work are deposited in the Genome Sequence Archive in National Genomics Data Center. The other datasets analyzed in the present work are available from the corresponding author upon reasonable request.

## Abbreviations

ALB: albumin
AST: aspartate aminotransferase
ALT: alanine transaminase
MVI: micro-vascular invasion
CI: confidence interval
HR: hazard ratio
CNV: copy number variation
C-index: concordance index
DAPI: 4',6-diamidino-2-phenylindole
DEG: differentially expressed gene
DAB: 3,3'-diaminobenzidine
DC: dendritic cell
EC: endothelial cell
FFPE: formalin-fixed paraffin-embedded
KEGG: kyoto encyclopedia of genes and genomes
GO: gene ontology
EMT: epithelial-mesenchymal transition
Grad-CAM: gradient-weighted class activation mapping
GSVA: gene set variation analysis
HCC: hepatocellular carcinoma
H&E: hematoxylin and eosin
HRP: horseradish peroxidase
IF: immunofluorescence
IHC: immunohistochemistry
L-TME: leading-edge tumor microenvironment
LSEC: liver sinusoidal endothelial cell
mIF: multiplex immunofluorescence
MVI: microvascular invasion
PCA: principal component analysis
UMAP: uniform manifold approximation and projection
PBS: phosphate-buffered saline
OCT: optimal cutting temperature compound
PVTT: portal vein tumor thrombus
RSF: random survival forest
SHAP: shapley additive explanations
scRNA-seq: single-cell RNA sequencing
snRNA-seq: single-nucleus RNA sequencing
TCGA: the cancer genome atlas
ST: spatial transcriptomics
STMN1: stathmin 1
TEC: tumor-associated endothelial cell
TLE: tumor leading edge
TME: tumor microenvironment
CAF: cancer-associated fibroblast
Treg: regulatory T cell
VSMC: vascular smooth muscle cell
WSI: whole-slide image
