# Supplementary material for: Spatial, single-nucleus and pathological profiling of the invasive front in early hepatocellular carcinoma for characterizing specific leading-edge cell niche and improving recurrence modeling
Source: Int J Biol Sci. 2026 Sep 10;22(14):8090–118. doi: 10.7150/ijbs.137262 (PMC13618224; doi:10.7150/ijbs.137262)
Supplement: Supplementary file 1 [file ijbsv22p8090s1.pdf]

Supplementary files

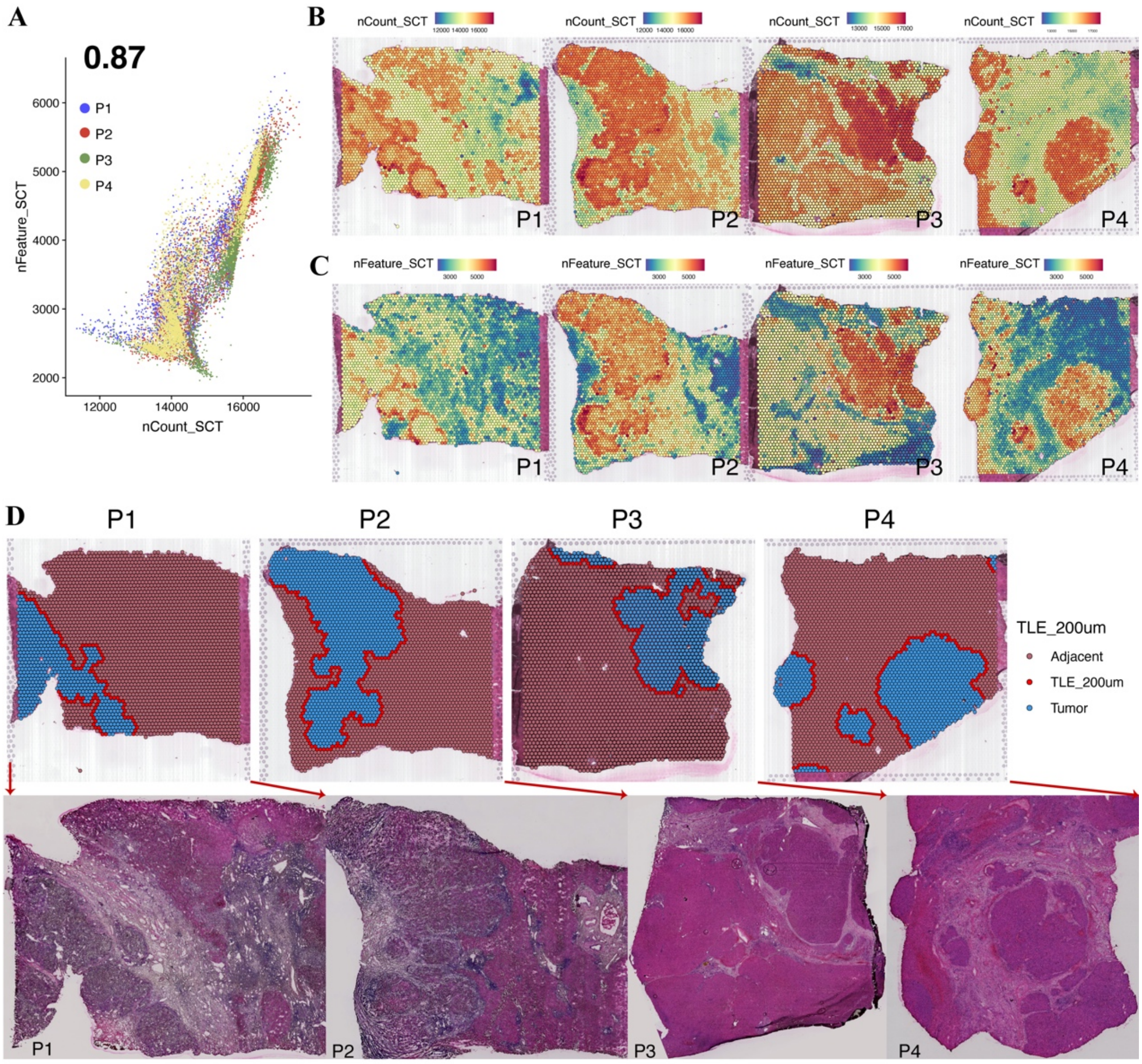

Figure S1. Batch effects were removed using stringent criteria and SCT methods. (A) Correlation analysis between SCT-standardized feature numbers and SCT-standardized UMI counts. (B) Spatial distribution of SCT-standardized UMI counts. (C) Spatial distribution of SCT-standardized feature numbers. (D) Spatial distribution of spatial regions. All 13546 spots colored by four early HCC leading-edge tissues were shown based spatial transcriptomics. Bright field microscope section images of four early HCC leading-edge tissues.

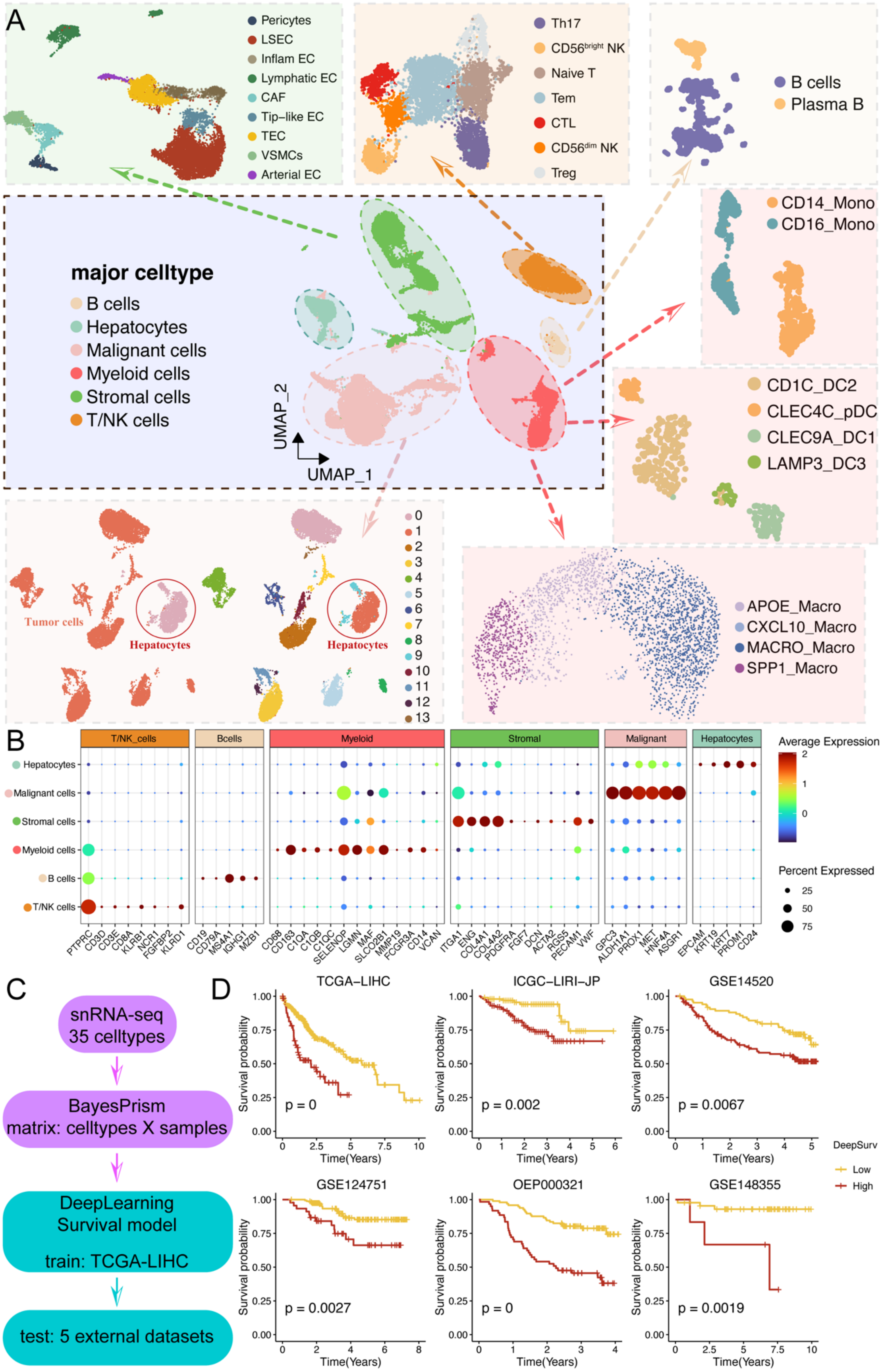

Figure S2. Subpopulation annotation of the detected major cell types and Construction of a prognostic model based on identified cell types in snRNA-seq

data. (A) There were total six major cell types found in the snRNA-seq data. And each major cell type could be further divided into specified subpopulations. (B) Dot plot shows the expression of markers of the major six major cell types. (C) Construction of a L-TME-based recurrence predictive model based on identified celltypes from snRNA-seq. Deep learning model was utilized. (D) The prognostic model was validated in other five HCC datasets, including ICGC-LIRI-JP, GSE14520, GSE124751, OEP000321 and GSE148355.

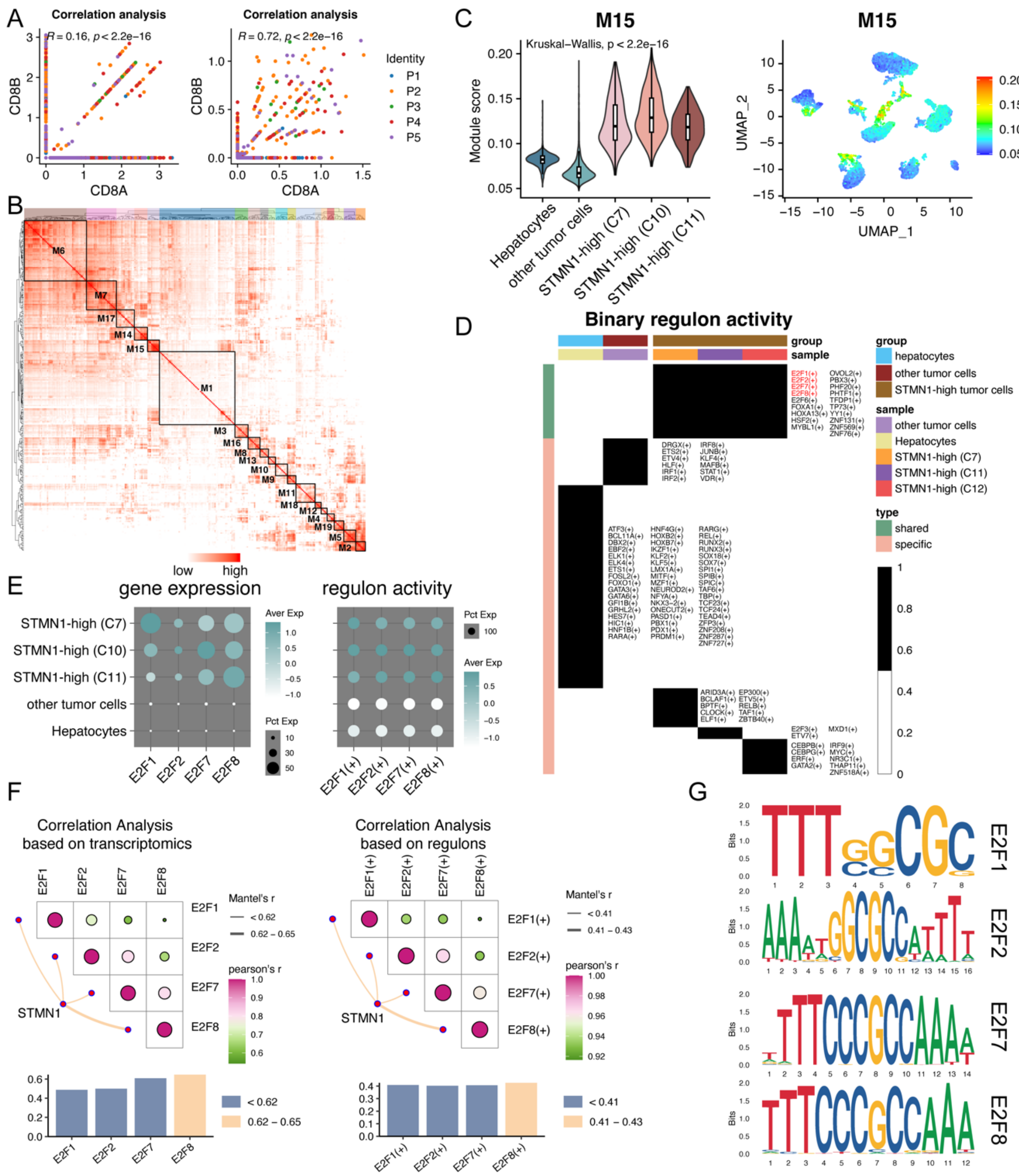

Figure S3. Transcription factor analysis of parenchymal cells, including hepatocytes and tumor cells. (A) Correlation analysis of CD8A and CD8B in the scRNA-seq data and meta-cell data, respectively. Obviously, the correlation coefficient was higher using meta-cell data, indicating that meta-cell data could reduce the potential dropout caused by scRNA-seq. (B) Regulon modules inferred by SCENIC pipeline. 19 Modules were obtained. (C) Only Module 15 was shown, which was elevated and activated in STMN1-high tumor cells. (D) Binary regulon activities were displayed using the heatmap plot. E2F families were enriched in STMN1-high tumor

cells. (E) Dot plot shows the gene expression levels and regulon activities of E2F families. (F) Correlation analysis among STMN1 and E2F families based on both gene expression levels and regulon activities. (G) The potential motifs of E2F1, E2F2, E2F7 and E2F8.

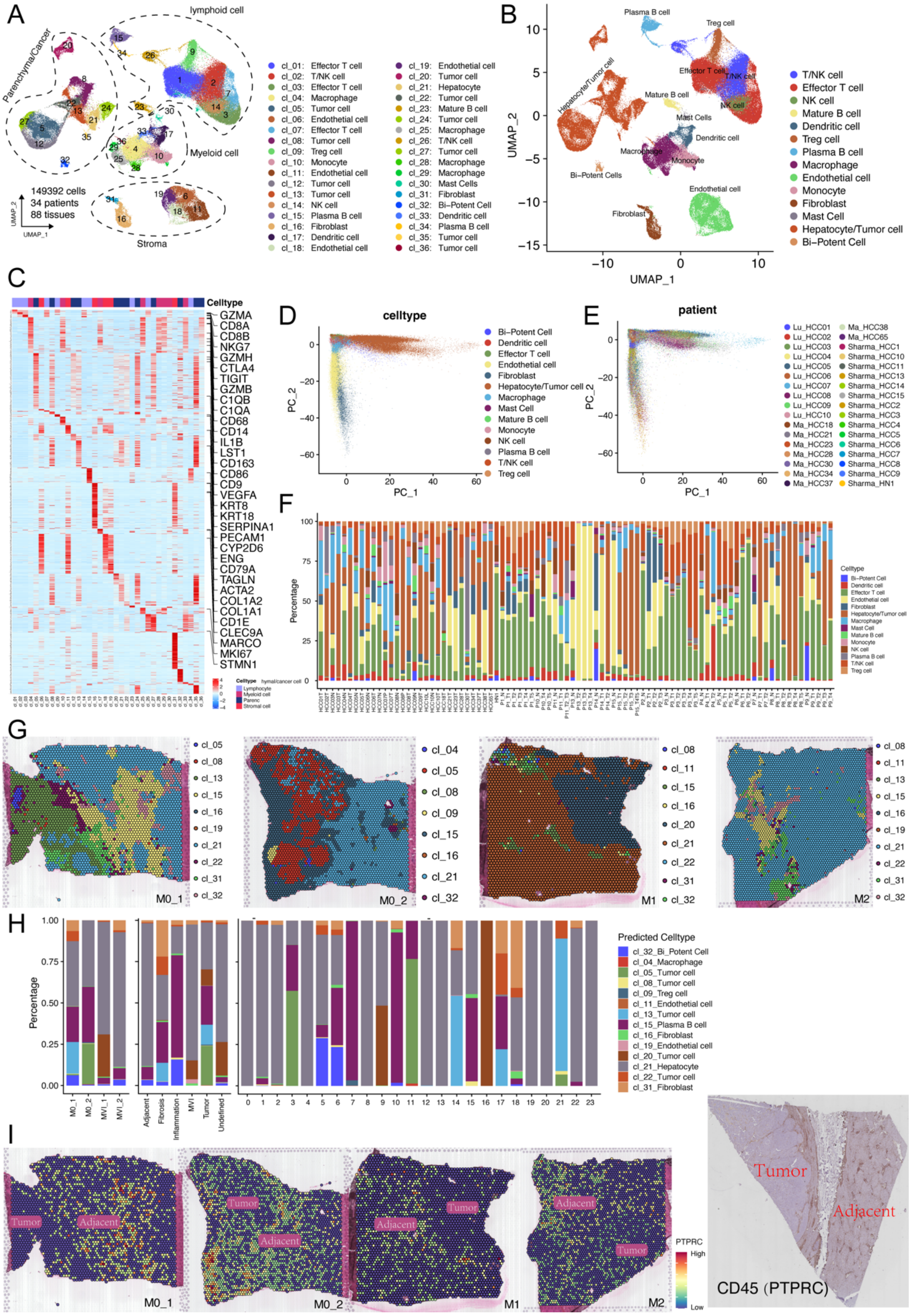

Figure S4. Combined analysis of spatial and single-cell transcriptomics data. (A) 149392 cells from 88 tissues of 34 HCC patients from three different cohorts were divided into 36 clusters, and separated into 4 major subpopulations. (B) UMAP plot of 149392 cells colored by 14 detailed celltypes. (C) Heatmap of celltype-related gene

expression. (D) PCA analysis of 14 celltypes. (E) PCA analysis of 34 HCC patients. (F) Proportion of 14 celltypes in each HCC patients. (G) Spatial distributions of predicted celltypes. (H) Proportion of predicted celltypes in each early HCC leading-edge tissues, spatial regions and spot clusters, respectively. (I) Spatial distribution of CD45 (PTPRC) (left); IHC image of CD45 (PTPRC) (right). Scale bar: 1mm.

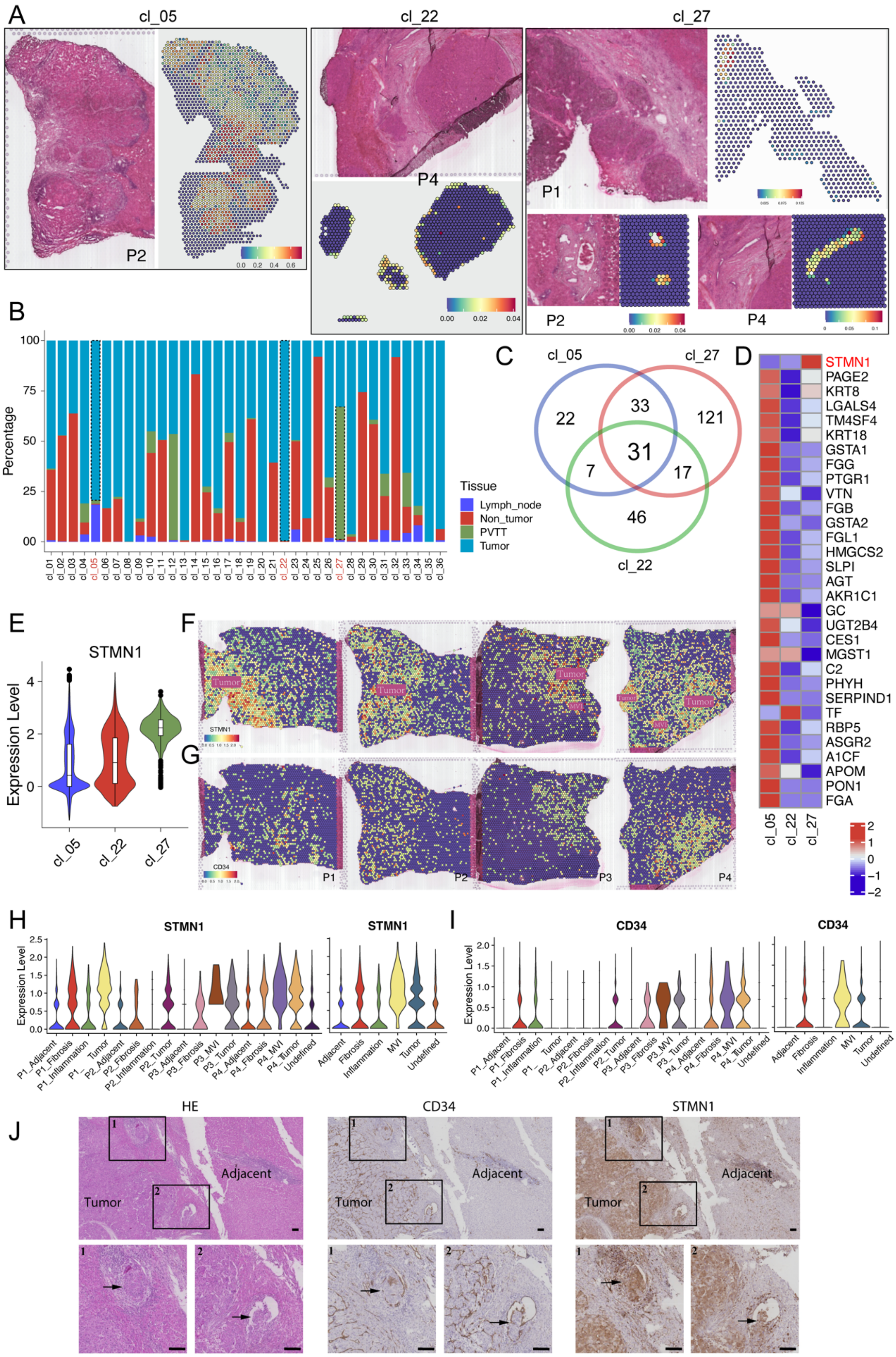

Figure S5. STMN1-high tumor cells contribute to the vascular invasion process of HCC. (A) Spatial distributions and the corresponding bright field microscope section

images of three predicted cell cluster, respectively. (B) Proportion of all single-cell clusters in different tissues. (C) Venn diagram of differentially expressed genes in three region-dependentcell clusters. (D) Heatmap of differentially expressed genes in three region-dependent clusters. (E) Violin plot of STMN1 expression in these three cell clusters. (F) Spatial distribution of STMN1 expression. (G) Spatial distribution of CD34 expression. (H) Violin plots of STMN1 expression in different regions of early HCC leading-edge tissues. Adjacent regions were further sub-classified into "Fibrosis", "Inflammation", "Adjacent" and "undefined" regions. Tumor regions were further denoted as "Tumor" and "MVI" regions. (I) Violin plots of CD34 expression in different area of early HCC leading-edge tissues. (J) HE staining image (left); IHC image of CD34 (middle); IHC image of STMN1 (right). J1) Tumor cells have invaded the micro-vessels, J2) Tumor cells are invading the micro-vessels. Scale bar: 100 μm.

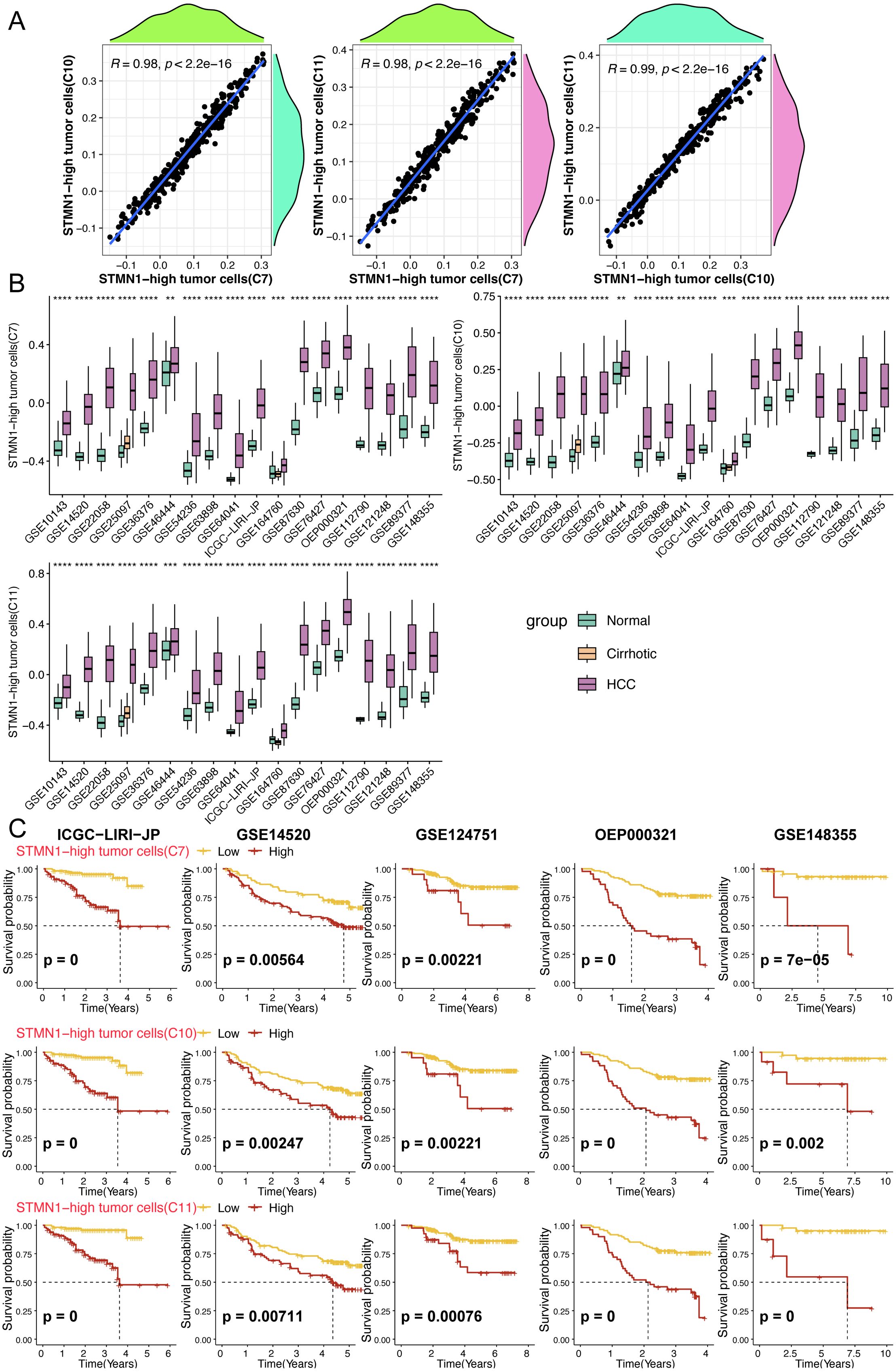

Figure S6. Comparison analysis of three STMN1-high tumor cells (Cluster 7, 10 and 11). (A) Correlation analysis of the abundance of these STMN1-high tumor cells

(Cluster 7, 10 and 11), related to Figure 2G and 2K. (B) The abundance comparisons between tumor and non-tumor tissues of these STMN1-high tumor cells (Cluster 7, 10 and 11), other tumor cells and hepatocytes in TCGA-LIHC database, related to Figure 2H. (C) Survival analysis of STMN1-high tumor cells (Cluster 7, 10 and 11) in another five datasets with survival information, related to Figure 2L. The three survival curves appear similar because the high-/low-risk group assignments based on the three STMN1-high subpopulations (C7, C10 and C11) were highly overlapping due to their correlated abundances.

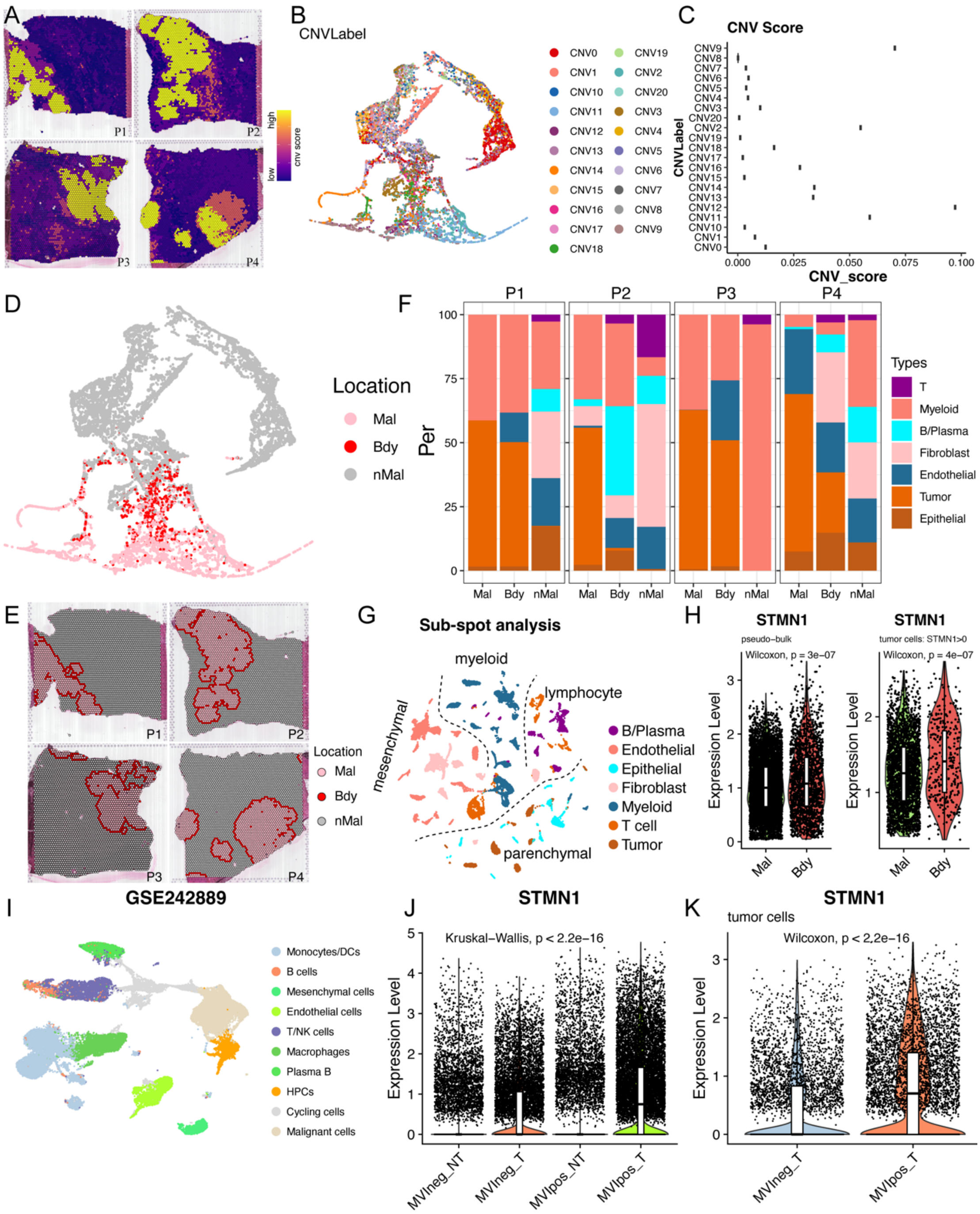

Figure S7. CNV analysis for the spatial transcriptomics. (A) CNV scores for all ST spots from 4 leading-edge slides. Cottrazm (Construction of Tumor Transition Zone Microenvironment) package was adopted for calculating CNV scores. (B) CNV clusters for all ST spots from 4 leading-edge slides. (C) Distribution of the cnv scores of all CNV clusters. (D)(E) Identification of the malignant, boundary (Bdy) and nonmalignant spots. Cottrazm package was adopted for reconstructing the malignant, boundary and nonmalignant spots based on CNV results. The predicted boundary spots

were identified and used here using Cottrazm workflow without manual selection, for avoiding potential preference. (F) Percentage of major cell types in the malignant, boundary and nonmalignant spots. The results of cell type deconvolution were consistent with that of identification of the malignant, boundary and nonmalignant spots. (G) Sub-spot (pseudo-single cell RNA) analysis based on Cottrazm package. (H) STMN1 expression in the Sub-spot single cell results. (I) Single cell RNA-seq analysis of HCC from GSE242889. (J) STMN1 expression of pseudo-bulk of scRNA-seq data. (K) STMN1 expression of pseudo-bulk of tumor cells in scRNA-seq data.

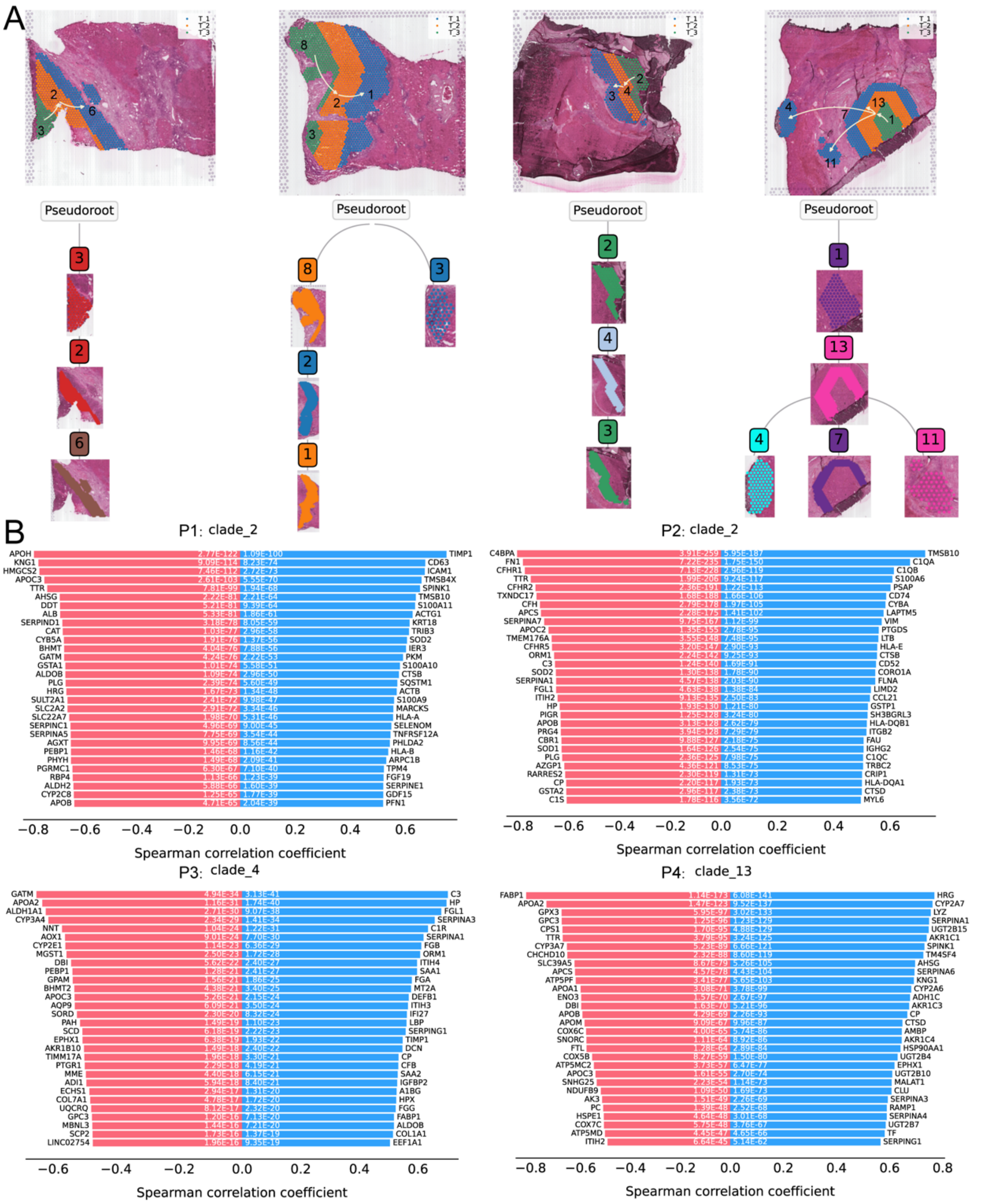

Figure S8. Pseudotime analysis of spatial transcriptomics. (A) Pseudotime analysis of spatial transcriptomics for 4 leading-edge slides. Stlearn python package was leveraged for analyzing the pseudotime changes. (B) Pseudotime-related genes for 4 leading-edge slides.

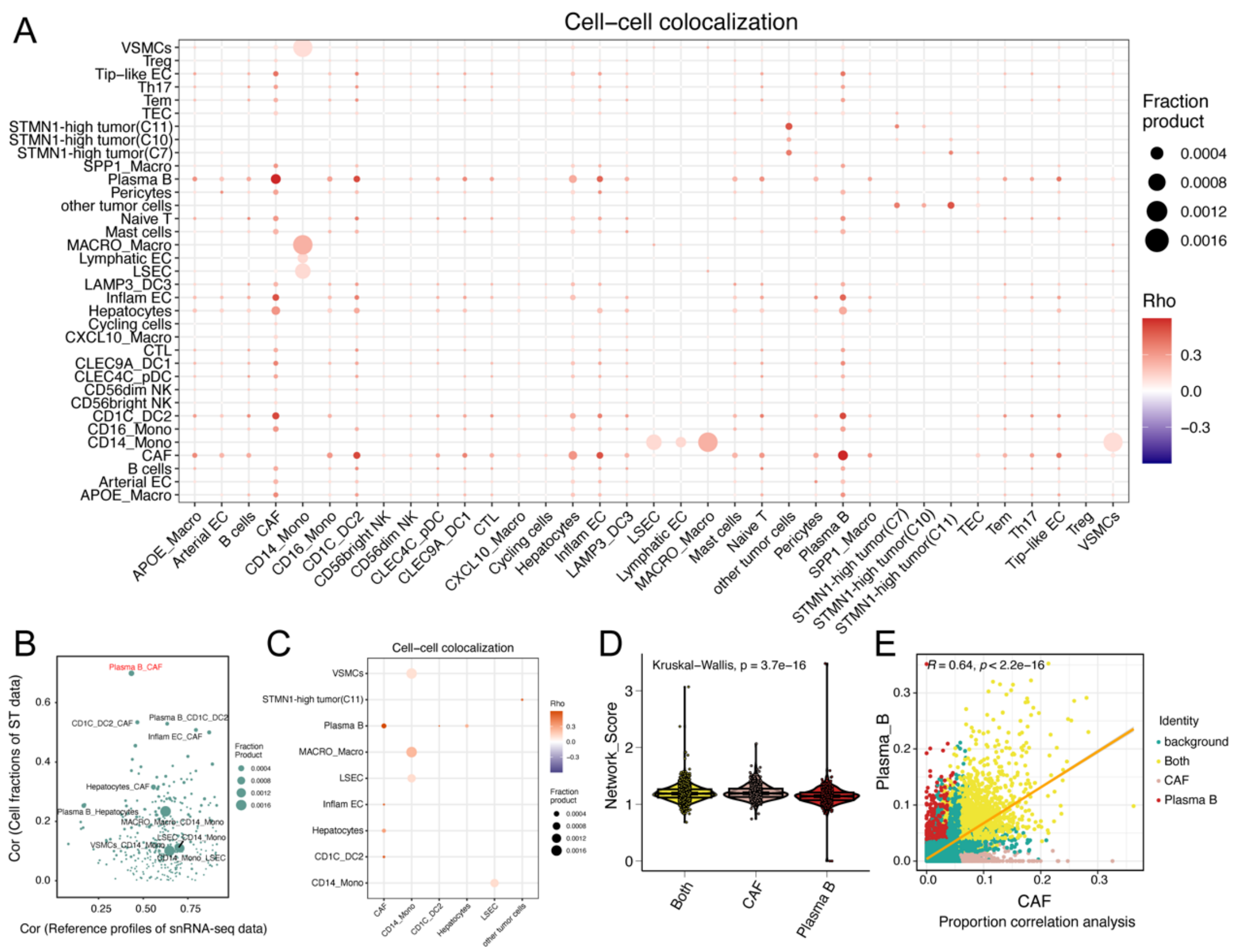

Figure S9. Spatial transcriptomics analysis for plasma B cells. (A) Cell-cell co-localization analysis of all identified cell types in snRNA-seq. (B) Scatter plot shows the correlation between the cell fraction of ST data and cell profiles of snRNA-seq data. The deconvolution results of ST data were calculated using SpaCET package (the Spatial Cellular Estimator for Tumors). (C) Cell-cell co-localization analysis and only significant results of cell-cell co-localization were shown. Rho: correlation coefficient. (D) Network score of three annotated spots: CAF spots, plasma B spots and CAF-plasma B (both) spots. (E) Correlation analysis of the proportion of plasma B cells and CAF.

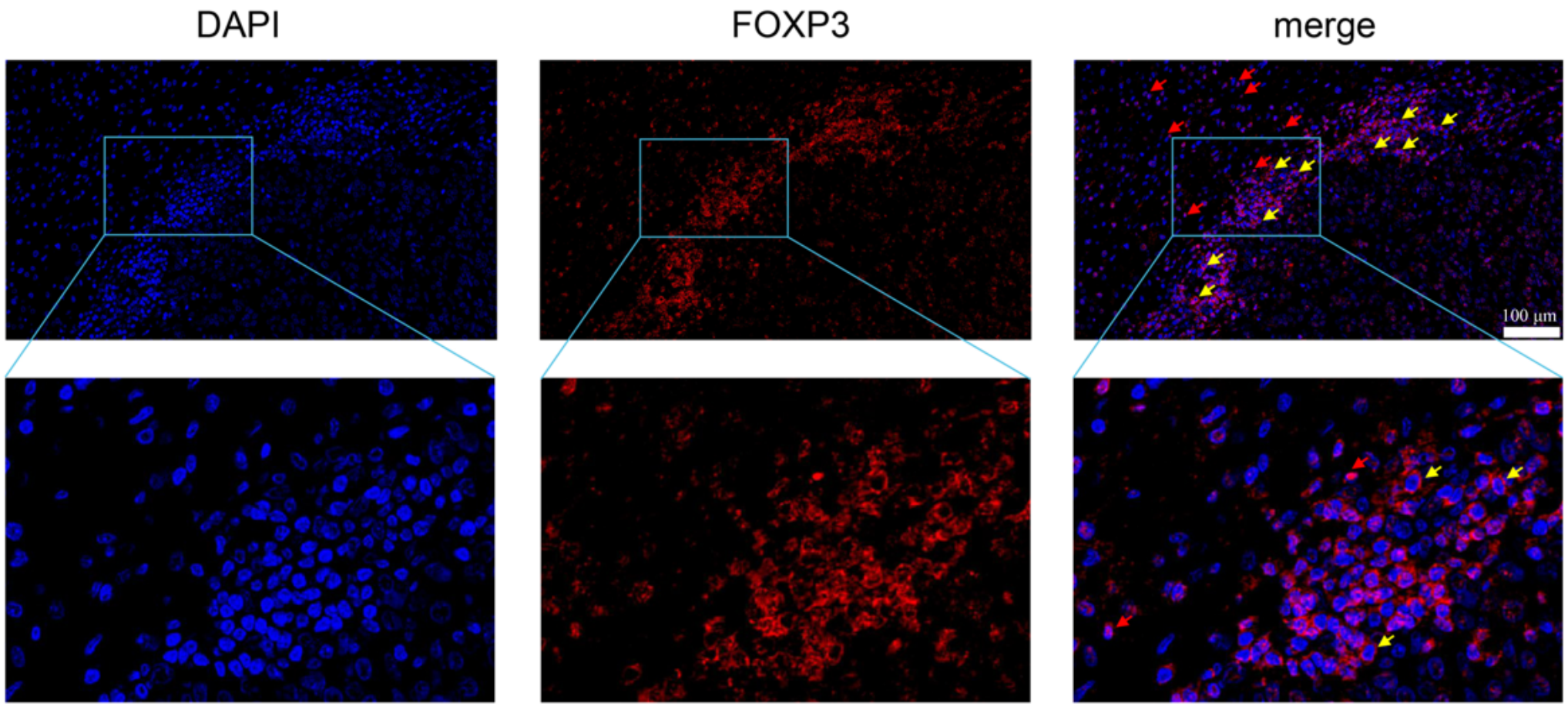

Figure S10. Immunofluorescence staining pattern of FOXP3 in Treg cells and tumor cells. The IF assay showing the merge of DAPI and FOXP3 was as follows: the red arrows represent the $CD3^{+}FOXP3^{+}$ Treg cells and the yellow arrows indicate the tumor cells expressing FOXP3. The detailed multiplex immunofluorescence staining results of CD3 and STMN1 were shown in Figure 4L.

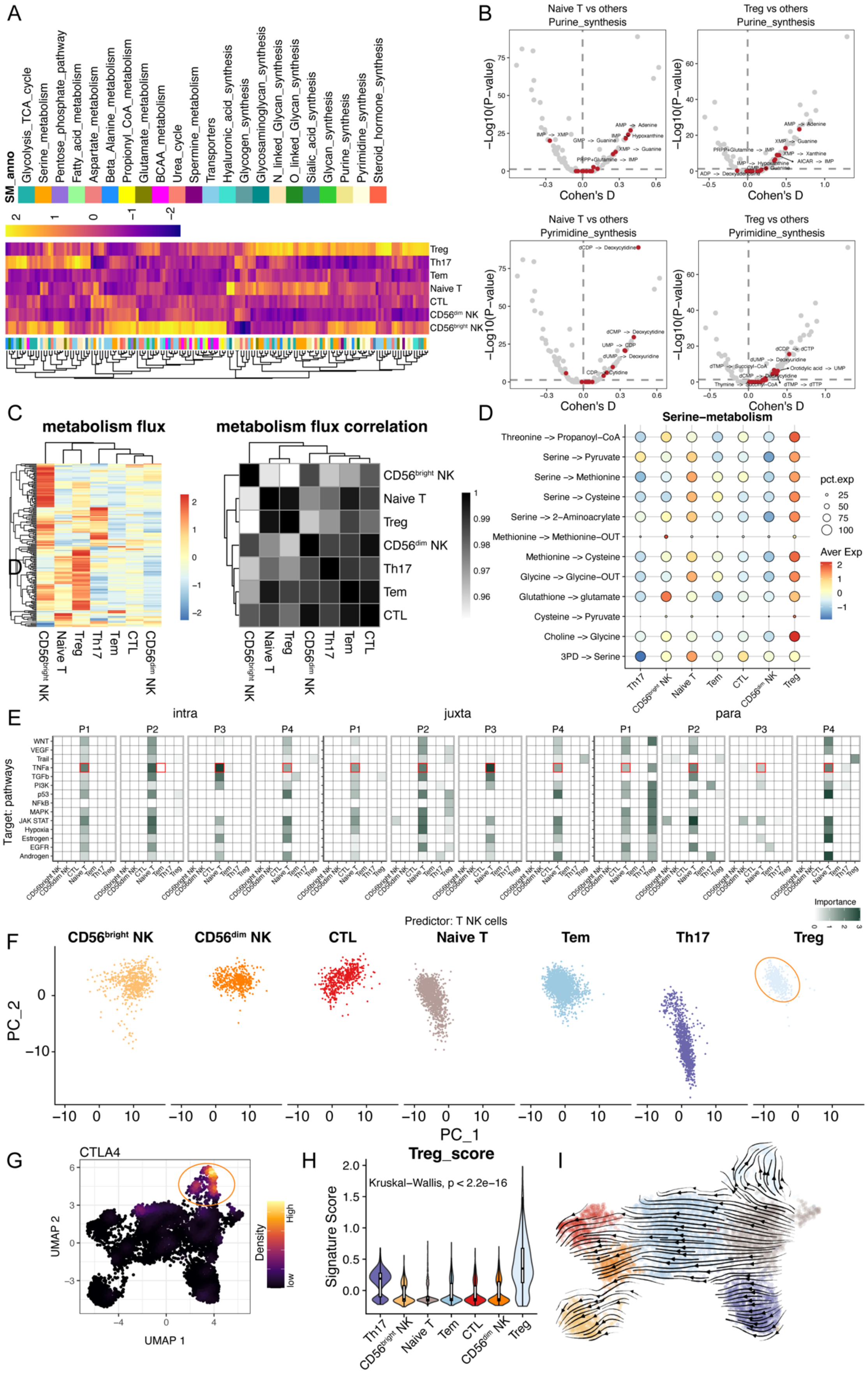
A
SM_anno
Glycolysis_TCA_cycle
Serine_metabolism
Pentose_phosphate_pathway
Fatty_acid_metabolism
Aspartate_metabolism
Beta_Alanine_metabolism
Propionyl_CoA_metabolism
Glutamate_metabolism
BCAA_metabolism
Urea_cycle
Spermine_metabolism
Transporters
Hyaluronic_acid_synthesis
Glycogen_synthesis
Glycosaminoglycan_synthesis
N_linked_Glycan_synthesis
O_linked_Glycan_synthesis
Sialic_acid_synthesis
Glycan_synthesis
Purine_synthesis
Pyrimidine_synthesis
Steroid_hormone_synthesis
Treg
Th17
Tem
Naive T
CTL
CD56dim NK
CD56bright NK
B
Naive T vs others
Purine_synthesis
Treg vs others
Purine_synthesis
Naive T vs others
Pyrimidine_synthesis
Treg vs others
Pyrimidine_synthesis
−Log10(P−value)
Cohen's D
C
metabolism flux
metabolism flux correlation
D
Serine−metabolism
Threonine −> Propanoyl−CoA
Serine −> Pyruvate
Serine −> Methionine
Serine −> Cysteine
Serine −> 2−Aminoacrylate
Methionine −> Methionine−OUT
Methionine −> Cysteine
Glycine −> Glycine−OUT
Glutathione −> glutamate
Cysteine −> Pyruvate
Choline −> Glycine
3PD −> Serine
pct.exp
Aver Exp
E
intra
juxta
para
P1
P2
P3
P4
Target: pathways
WNT
VEGF
Trail
TNFa
TGFb
PI3K
p53
NFkB
MAPK
JAK STAT
Hypoxia
Estrogen
EGFR
Androgen
Predictor: T NK cells
Importance
F
CD56bright NK
CD56dim NK
CTL
Naive T
Tem
Th17
Treg
PC_2
PC_1
G
CTLA4
UMAP 2
UMAP 1
Density
High
low
H
Treg_score
Kruskal−Wallis, p < 2.2e−16
Signature Score
Th17
CD56bright NK
Naive T
Tem
CTL
CD56dim NK
Treg
I

Figure S11. Metabolic and functional analyses of T/NK cells. (A) Heatmap shows the metabolic flux among T/NK cells. ScFEA pipeline were performed in python to infer the metabolic flux of T/NK cells. (B) Volcano plot shows the metabolic changes of Treg and Naive T cells versus other tumor cells, respectively. (C) Clustering analysis of T/NK cells based on metabolic flux and metabolic genes, respectively. Treg and Naive T cells were clustered together. (D) Metabolic flux of serine metabolism in all T/NK cells. The metabolism flux of serine metabolism in Treg and Naive T cells was similar. (E) Activities of crucial pathways in all T/NK cells based on mistyR and Progeny package. (F) PCA pipeline for T cells. (G) UMAP plot shows the expression distribution of CTLA4, a canonical biomarker of Treg cells. (H) Treg cell score was estimated using "AddModule" function in Seurat package. (I) Trajectory analysis of T cells. The pseudotime was calculated by scTour package in python, a deep learning model for robust prediction of cell dynamics.

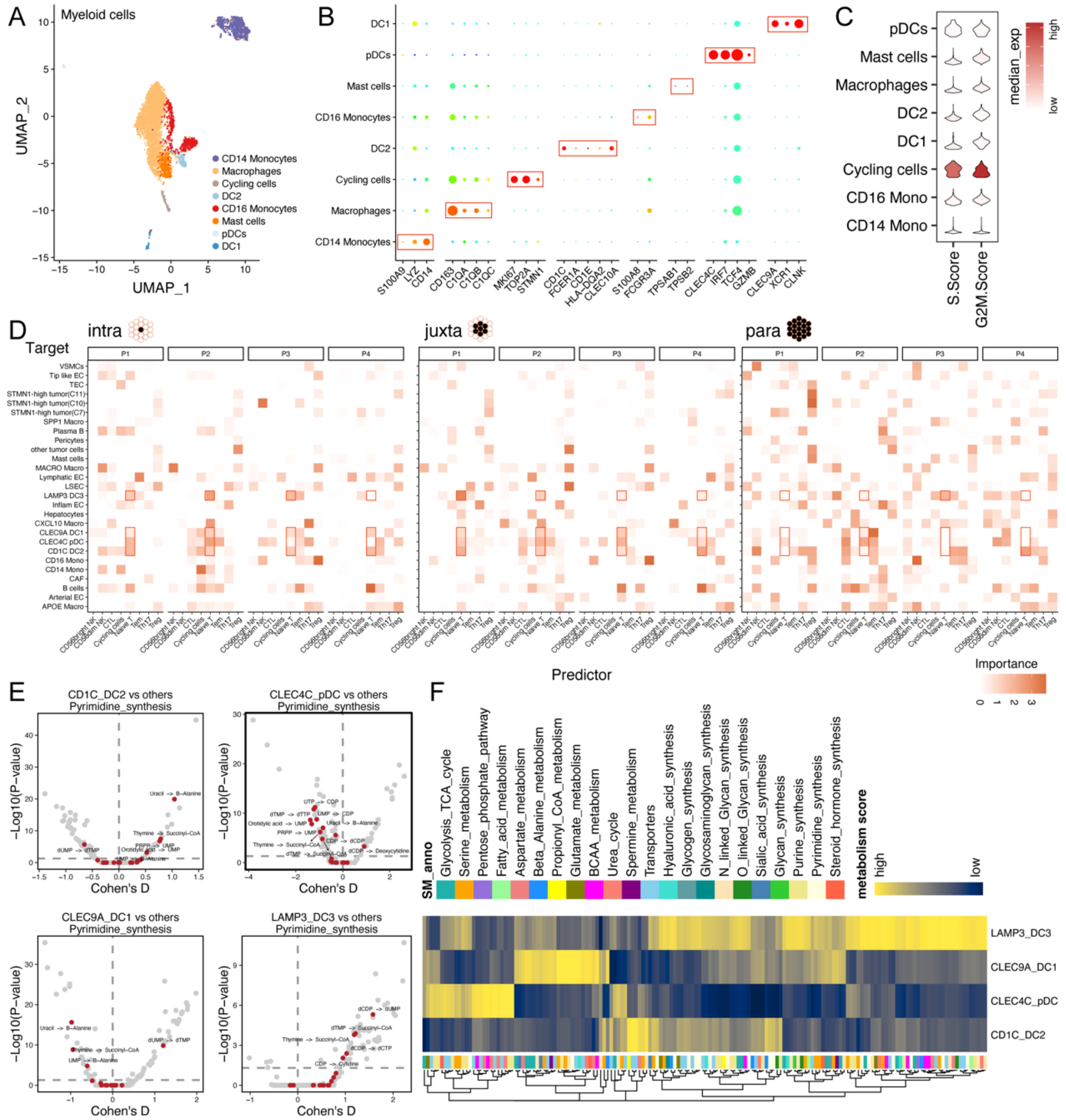

Figure S12. Metabolic analysis of myeloid cells, especially dendritic cells. (A) UMAP plot displays the cell distribution of myeloid cells. (B) Expression levels of Biomarkers of myeloid cells. (C) Cell cycle scores of all myeloid cells based on AddModule algorithm. (D) Cell-cell co-localization analysis of all other cell types with T/NK cells. The dendritic cells were highlighted. (E) Volcano plot shows the metabolic changes of the four identified dendritic cells. ScFEA pipeline was performed in python to infer the metabolic flux of dendritic cells. (F) Heatmap shows the metabolic flux among four identified dendritic cells. Most metabolic fluxes in LAMP3^{+} dendritic cells were activated.

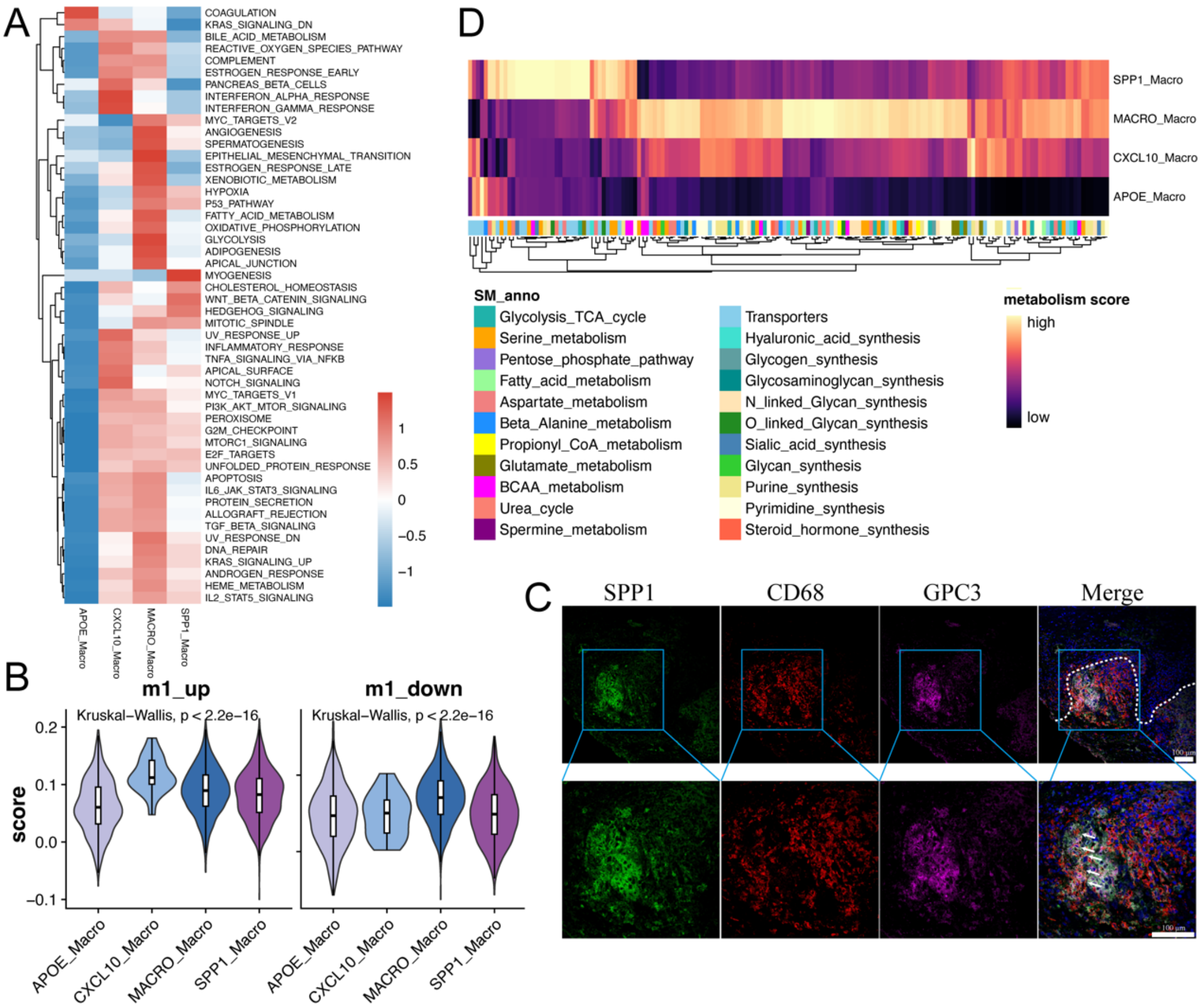

Figure S13. Single nucleus RNA-seq analysis of macrophages. (A) Pathway scores of hallmark pathway terms of the four macrophages based on GSVA algorithm. (B) M1 and M2 score levels in four macrophages, estimated by "AddModule" algorithm. (C) Immunofluorescence assays for SPP1 macrophages and tumor cells. (D) Heatmap shows the metabolic flux among four identified macrophages. Most metabolic fluxes in four types of macrophages were quite distinct.

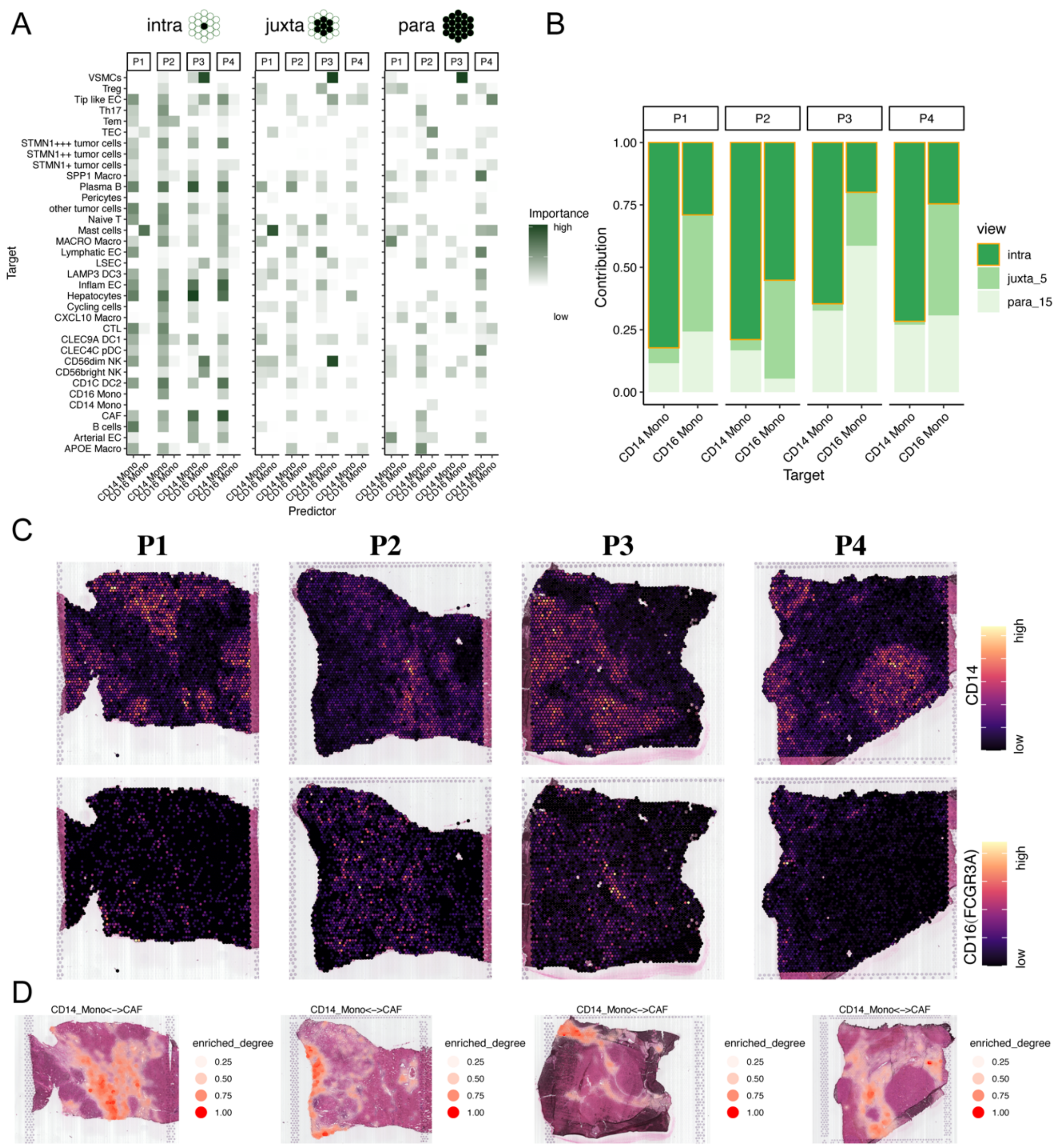

Figure S14. Spatial transcriptomics analysis for monocytes. (A) Cell-cell co-localization analysis of all cell types with CD14 and CD16^{+} monocytes. The cell-cell co-localization analysis was performed using mistyR. (B) Cell contribution distribution for intra-, juxta- and para-spots, respectively. (C) Expression levels of CD14 and CD16 in spatial transcriptomic data. (D) Enriched degree of CD14^{+} monocytes with CAF.

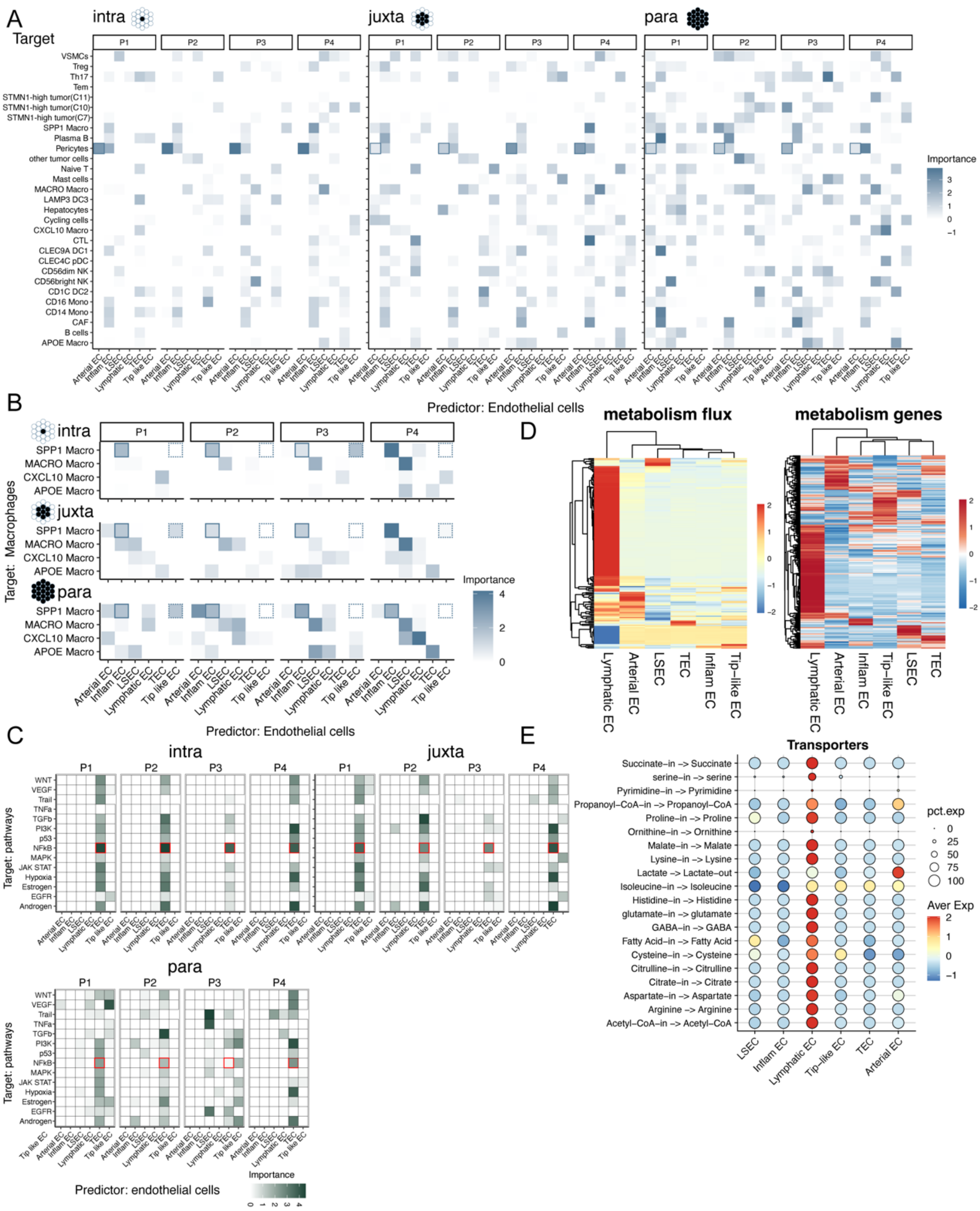

Figure S15. Spatial transcriptomics analysis for endothelial cells. (A) Cell-cell co-localization analysis of all cell types with endothelial cells. The cell-cell co-localization analysis was performed using mistyR. (B) Cell-cell co-localization analysis of macrophages with endothelial cells. SPP1^{+} macrophages were highlighted. (C) Enriched pathways of endothelial cells based on spatial transcriptomic data. The misty R package was used. (D) Clustering analysis of the sub-clusters of endothelial cells based on metabolic fluxes and metabolic genes. (E) Metabolic flux of transports co-

metabolism in endothelial cells. The metabolism flux of transports in lymphatic endothelial cells was highest compared to other endothelial cells.

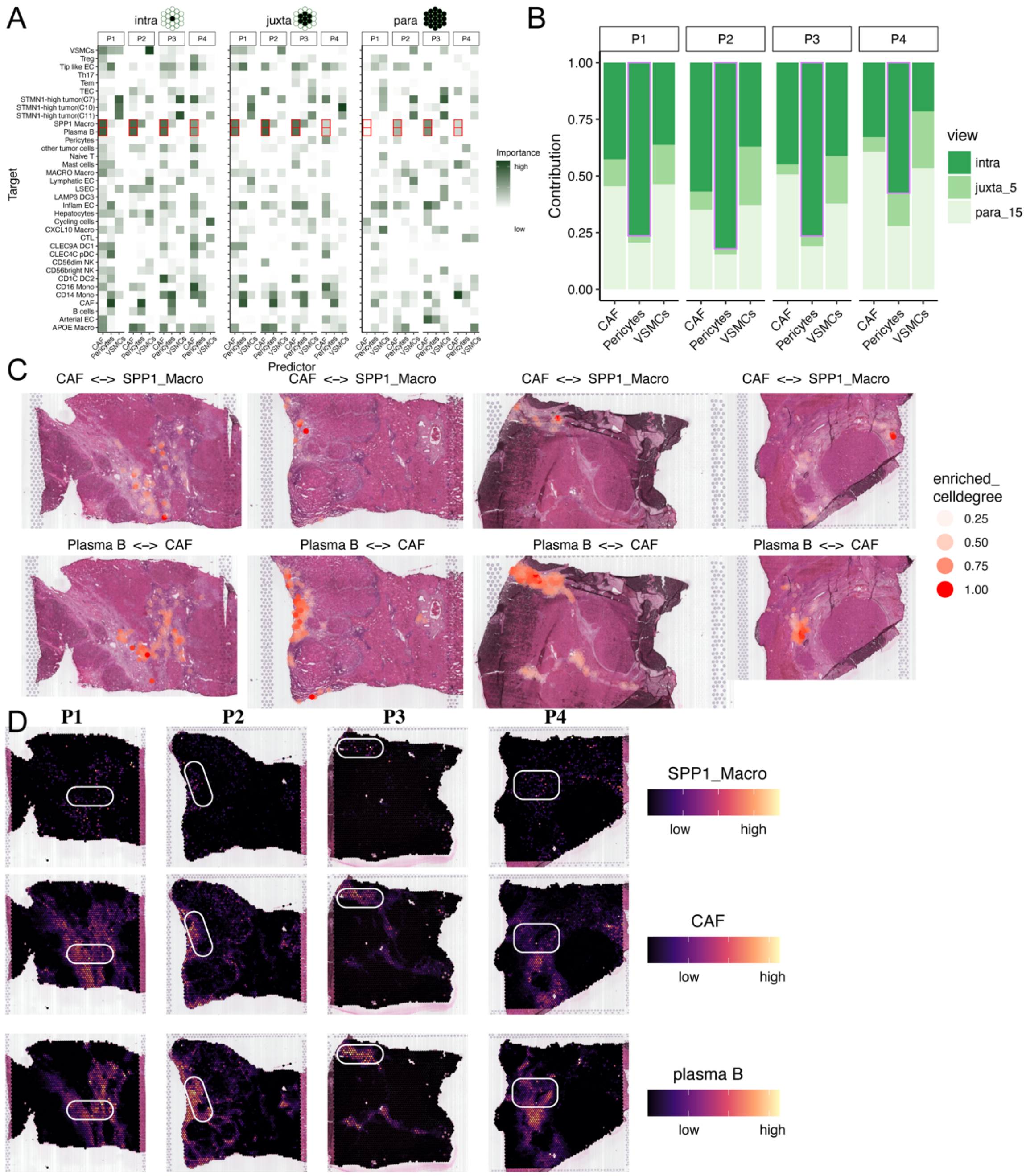

Figure S16. Spatial transcriptomics analysis for stromal cells. (A) Cell-cell co-localization analysis of all cell types with stromal cells. The cell-cell co-localization analysis was conducted by using mistyR. (B) Cell contribution distribution for intra-, juxta- and para-spots, respectively. (C) Enriched cell degree of SPP1^{+} macrophages

with CAF, and plasma B cells with CAF. (D) Spatial distribution of $SPP1^+$ macrophages, plasma B cells and CAF. The potential co-localization areas were underlined.

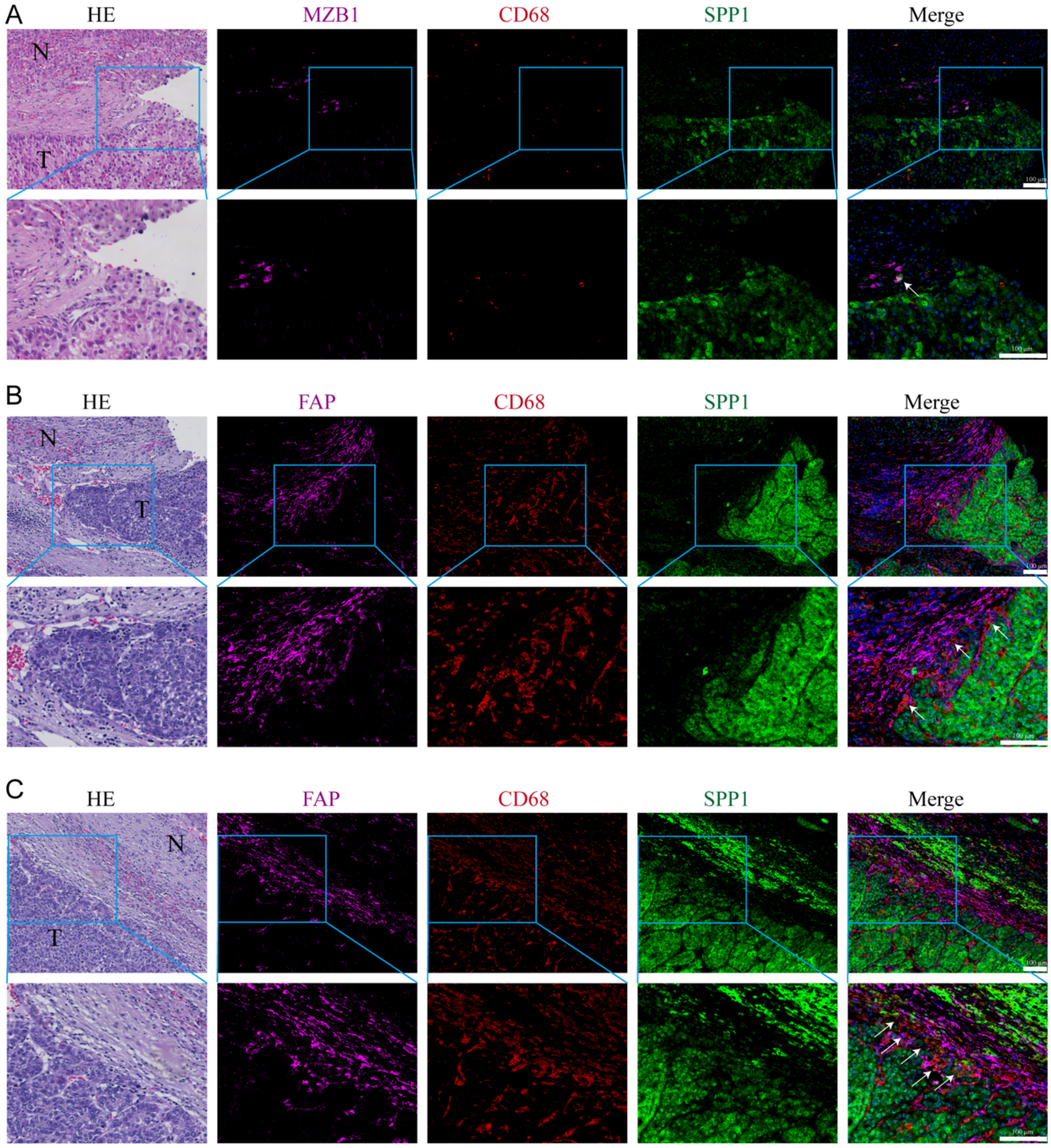

Figure S17. Immunofluorescence assays for validating co-localization results. (A) Immunofluorescence assays for $SPP1^+$ macrophages and plasma B cells. (B) Immunofluorescence assays for $SPP1^+$ macrophages and CAF. (C) Immunofluorescence assays for $SPP1^+$ macrophages and CAF in liver tissues of another early HCC patient.

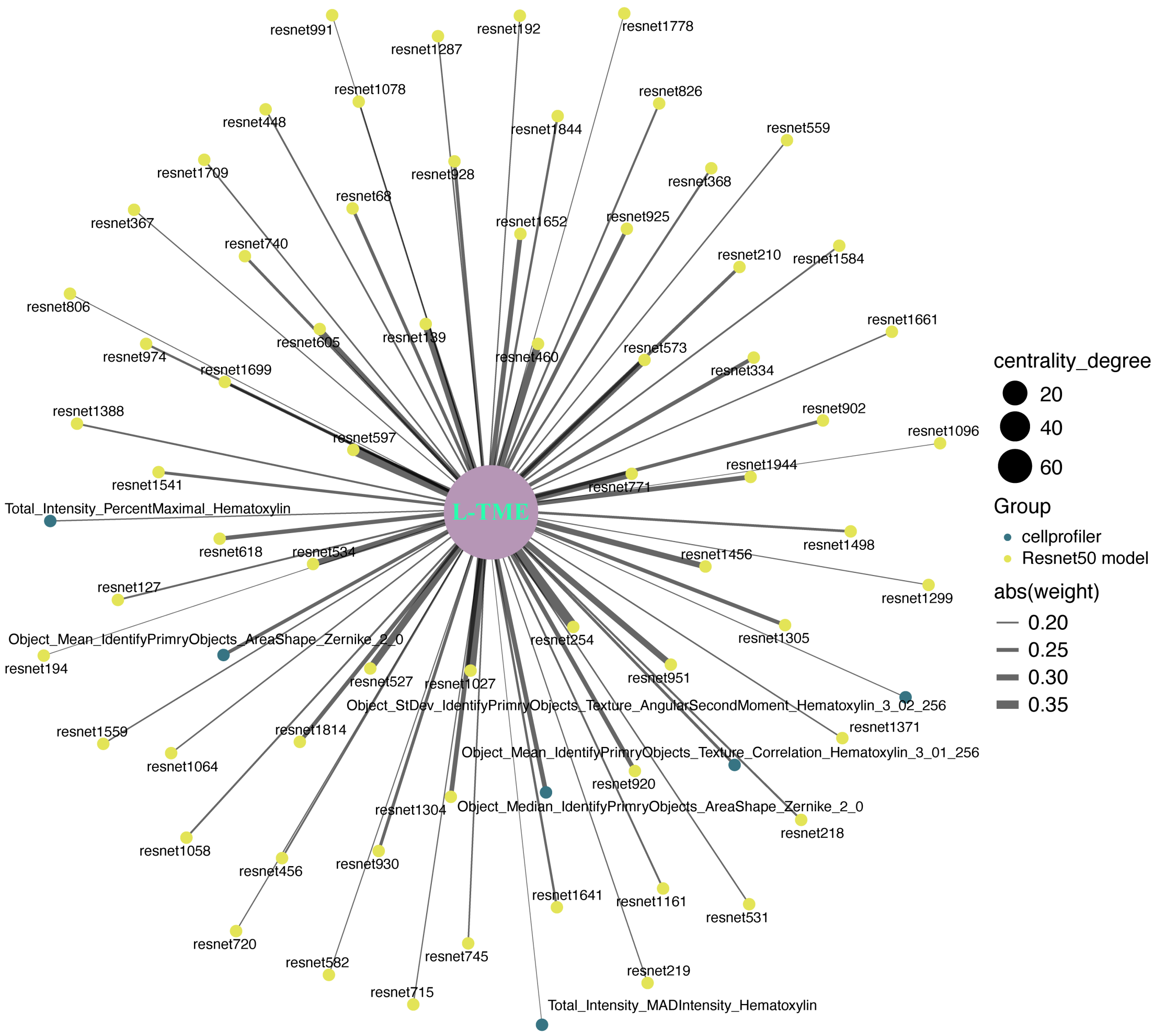

Figure S18. Correlation analysis between L-TME niche and pathological features.

Only the results with significant correlation coefficient were shown.

Table S1. Definitions of tumor leading-edge region (TLE) in different studies.

Names	Teams	Definition<br>(tumor to adjacent tissues)	Referen<br>ces
leading-edge area/<br>transitional zone	Zheng et al.	-(0.4-0.6cm) to<br>+ (0.4-0.6cm)	[1]
invasive margin (rim)	Ruf et al.	-0.5cm to +0.5cm	[2]
margin area	Shi et al.	-500μm to +500 μm	[3]
invasive margin	Halama et al.	-500μm to +500 μm	[4]

leading-edge area	Wu et al.	-(750-250μm) to +(750-250 μm)	[5]
invasive zone	Wu et al.	-250μm to +250μm	[5]
TLE_200μm: Boundary of "tumor-to-adjacent" interface of the ST data from 10× Visium platform	Our present study	-100μm to +100μm	Our present study
Sample collection of snRNA-seq data	Our present study	-(0.4-0.6cm) to + (0.4-0.6cm)	Our present study

Table S2. Clinical and Pathological Information of Each patient.

Patient ID	P1	P2	P3	P4	P5
Sequencing	Spatial transcriptomics and snRNA sequencing				snRNA sequencing
Sex	Male	Male	Male	Male	Male
Age	40	60	69	50	65
Etiology of liver disease	HBV	HBV	HBV	HBV	HBV
AST/ALT	33/54	16/15	18/10	26/24	53/42
Cirrhosis	Yes	No	No	Yes	No
ALB (g/L)	42	46.1	39.6	40.4	48.2
AFP (ug/ml)	18.3	84	2.4	609	115
Ascites	0	0	0	0	0
Child-Pugh grade	A	A	A	A	A
BCLC Stage	A	A	A	A	A
Tumor size	3*3	2*1.9	2.2*2	4.5*4	4.8*4.5

MVI	M0	M0	M1	M2	M2
Type of Resection	R0	R0	R0	R0	R0
Tumor Stage	I	I	I	I	I
TNM Groups	T1N0M0	T1N0M0	T1N0M0	T1N0M0	T1N0M0

Abbreviations: ALT, Alanine transaminase; AST, Aspartate aminotransferase; ALB, Albumin; BCLC stage, Barcelona clinic liver cancer stage; MVI, Microscopic vascular invasion; TNM stage, Tumor node metastasis stage

Table S3. Primary antibodies used in the IHC and Immunofluorescence assay.

**Primary Antibody**	**Brand**	**Cat. No.**
Stathmin 1	Cell Sigaling Technology	3352
Stathmin 1	Abcam	ab52630
CD45	MXB Biotechnlogies	Kit-0024
MZB1	Proteintech	11454
XBP1	Abcam	ab37152
CD3	Abcam	ab135372
Foxp3	Abcam	ab215206
LAMP3	Cell Sigaling Technology	47778
CD68	Cell Sigaling Technology	76437
SPP1	Cell Sigaling Technology	88742
FAP	Cell Sigaling Technology	66562

References

1. Zheng B, Wang D, Qiu X, Luo G, Wu T, Yang S, et al. Trajectory and Functional Analysis of PD-1(high) CD4(+)CD8(+) T Cells in Hepatocellular Carcinoma by Single-Cell Cytometry and Transcriptome Sequencing. Adv Sci (Weinh). 2020; 7: 2000224.
2. Ruf B, Bruhns M, Babaei S, Kedei N, Ma L, Revsine M, et al. Tumor-associated macrophages trigger MAIT cell dysfunction at the HCC invasive margin. Cell. 2023; 186: 3686-705 e32.
3. Shi JY, Gao Q, Wang ZC, Zhou J, Wang XY, Min ZH, et al. Margin-infiltrating CD20(+) B cells display an atypical memory phenotype and correlate with favorable prognosis in hepatocellular carcinoma. Clin Cancer Res. 2013; 19: 5994-6005.
4. Halama N, Michel S, Kloor M, Zoernig I, Benner A, Spille A, et al. Localization and density of immune cells in the invasive margin of human colorectal cancer liver metastases are prognostic for response to chemotherapy. Cancer Res. 2011; 71: 5670-7.
5. Wu L, Yan J, Bai Y, Chen F, Zou X, Xu J, et al. An invasive zone in human liver cancer identified by Stereo-seq promotes hepatocyte-tumor cell crosstalk, local immunosuppression and tumor progression. Cell Res. 2023; 33: 585-603.
